# Recent developments in the cleavage, functionalization, and conjugation of proteins and peptides at tyrosine residues

**DOI:** 10.1039/d3sc02543h

**Published:** 2023-06-27

**Authors:** Shengping Zhang, Luis M. De Leon Rodriguez, Freda F. Li, Margaret A. Brimble

**Affiliations:** a Center for Translational Medicine, Shenzhen Bay Laboratory New Zealand; b School of Chemical Sciences, The University of Auckland 23 Symonds St Auckland 1010 New Zealand m.brimble@auckland.ac.nz; c School of Biological Sciences, The University of Auckland 3A Symonds St Auckland 1010 New Zealand; d Maurice Wilkins Centre for Molecular Biodiscovery, The University of Auckland 1142 New Zealand

## Abstract

Peptide and protein selective modification at tyrosine residues has become an exploding field of research as tyrosine constitutes a robust alternative to lysine and cysteine-targeted traditional peptide/protein modification protocols. This review offers a comprehensive summary of the latest advances in tyrosine-selective cleavage, functionalization, and conjugation of peptides and proteins from the past three years. This updated overview complements the extensive body of work on site-selective modification of peptides and proteins, which holds significant relevance across various disciplines, including chemical, biological, medical, and material sciences.

## Abstract 2

This review covers recent progress in tyrosine-selective cleavage, functionalization, and conjugation of peptides and proteins. Key applications of Tyr modification are highlighted, demonstrating its great potential for chemistry and biology.

## Graphical abstract

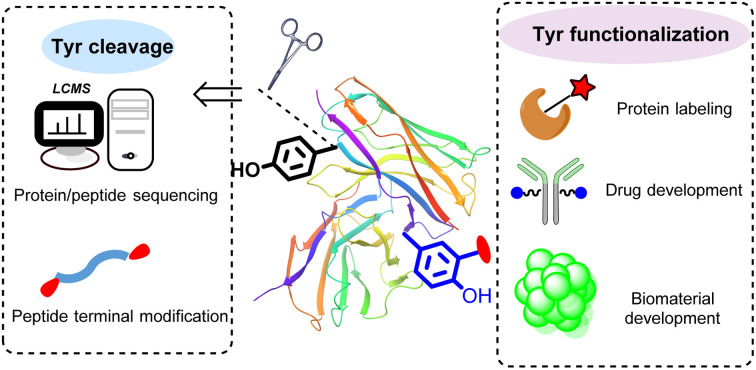

## 1 Introduction

Tyrosine (Tyr) is an amino acid that is essential to many biochemical processes. It is found in the active sites of numerous enzymes and plays an important role in protein–protein and protein-ligand interactions.^1,2^ The phenol functionality in the side chain of Tyr is amphiphilic, which allows it to contribute to both hydrophobic interactions through its aromatic ring and hydrogen bonding with binding partners *via* the hydroxyl group. Additionally, Tyr is susceptible to various post-translational modifications, such as glycosylation, nitration, oxidation, and phosphorylation, which highlights its importance in human disease states, such as Alzheimer's disease and cancer.^3–5^ Consequently, there is a growing need for tools that allow the investigation of the function of Tyr in proteins and bioactive peptides. Selective Tyr modification, achieved enzymatically, chemically, or by genetically encoded non-natural amino acids, and selective peptide cleavage at Tyr sites have emerged as relevant tools in this context.

Protein/peptide selective modification and conjugation is a rapidly-growing area that has demonstrated tremendous potential for applications in proteomics,^6^ drug optimization,^7^ targeted drug delivery,^8^ and defined biomaterials.^9^ Traditional methods focusing on selective Cys and Lys modifications have been well-documented and are widely used in various scenarios.^10^ However, the interest in Tyr as a key alternative target for protein modification and conjugation has been steadily increasing due to several unique properties of this amino acid. Tyr residues generally show low abundance on the surface of proteins and are partially or fully buried in the protein structure. Hence, each Tyr residue is expected to be embedded in a distinct microenvironment within the protein structure, thus rendering the region-selective protein modification achievable. Moreover, modification on Tyr has no impact on the net charge of proteins as Tyr remains in its neutral state under a wide range of pH conditions. Additionally, unlike protein conjugation *via* Cys which requires a pre-reduction step to liberate the free thiol group, Tyr is readily available for conjugation.^11^ In light of these advantages, the development of methods for selective tyrosine modification is of great value as a complement to the existing protein modification/conjugation strategies, especially in circumstances where Cys and Lys conjugation techniques are not applicable, or orthogonality is required to install different cargoes on the same protein.^12^

An elegant review was reported by Alvarez Dorta *et al.* in 2020, which summarized the chemical approaches utilized in the conjugation of Tyr over the last 15 years.^13^ Other related reviews covering periods before 2021 can also be found in the literature.^14–16^ Additionally, although not specifically focused on Tyr, several reviews provide general overviews of protein conjugation *via* canonical and non-canonical residues,^17–20^ as well as some centered on specific chemistries (*e.g.*, metal-mediated C–H functionalization, oxidation-induced click chemistry, *etc.*).^21–24^ The rapid advancements in tyrosine conjugation methods in recent years necessitate an up-to-date summary of the current state of this field. As such, this review will focus on the progress achieved within the last three years.

It is also noteworthy that site-selective cleavage of the amide bond in peptides and proteins is an essential chemical transformation that has found promising applications in proteomics^25^ and site-specific functionalization,^26,27^ a topic of high relevance in the design of novel therapeutics.^28^ To determine the sequence of an unknown peptide or protein substrate, enzymatic or chemical cleavage of peptide bonds at specific residues is typically followed by amino acid analysis or liquid chromatography with tandem mass spectrometry (LC-MS/MS).^29^ Biologists are interested in developing novel peptide-bond cleaving methods targeting different amino acids to ensure broad sequence coverage.^30^ Furthermore, chemical cleavage of the peptide bond usually results in the formation of modified C- or N-terminal peptide fragments, some of which are highly reactive and can be further derivatized to enable selective functionalization at peptide termini.^27,31^ Despite different methods having been disclosed for Tyr-selective cleavage of peptides/proteins over a 60 year timespan,^32–34^ there is no systematic review that covers this area.^35,36^ Hence, to fill this gap, a section of this review will address the latest updates on the selective peptide/protein cleavage at Tyr sites.

## 2 Amide bond selective cleavage at Tyr sites of proteins/peptides

Taking advantage of the electron-rich phenol side chain of tyrosine, peptide bond cleavage at Tyr residues can be accomplished through a range of methods, including chemical, electrochemical, and enzymatic approaches. These diverse strategies enable the selective cleavage of peptide bonds at tyrosine sites in peptides and proteins.

### 2.1 Chemical methods

**Fig. 1:**
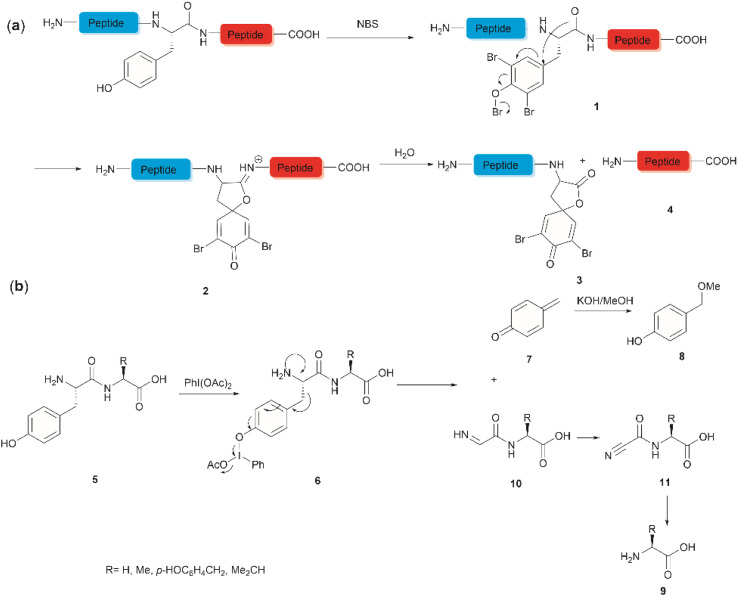
Tyr-selective peptide cleavage using (a) NBS and (b) PhI(OAc)_2_.

The development of chemical reagents that enable peptide bond scission at specific residues has long been of key interest for protein/peptide sequencing. In 1959, Cohen *et al.* reported the first selective peptide/protein cleavage at Tyr using *N*-bromosuccinimide (NBS).^33^ In this reaction, the peptide bond rupture occurred at the C-terminal amide of tyrosine, resulting in a modified N-terminal peptide fragment 3 featuring a spirodienone-lactone moiety, as well as an intact C-terminal peptide fragment 4. It was proposed that this process was triggered by oxidative bromination of the phenol ring by NBS, followed by lactonization of the C-terminal carbonyl of Tyr with the formed tri-bromophenol species 1 (Fig. 1a). The resulting carboximidate intermediate 2 within the peptide backbone was highly susceptible towards hydrolysis in the presence of water, thereby leading to facile peptide fragmentation at the Tyr site. This cleavage reaction was effectively carried out under acidic conditions (pH 4.6) at room temperature and demonstrated a wide substrate scope, encompassing both modified peptides and proteins.^33,37–39^ Despite these advantages, further application of the NBS-mediated peptide cleavage in peptide/protein sequencing was hindered by extensive modification of other amino acid sidechains (Met, Cys, and His), moderate yields and low site specificity as Trp was found to be a more favorable cleavage site than Tyr. Similarly, iodosuccinimide was also found to be capable of cleaving peptides at the C-terminal amide of Tyr.^40^

Hypervalent iodine compounds, such as iodine(iii) and iodine(v), are a group of prevailing organocatalysts used for diverse oxidative transformations as they are mild, non-toxic, water-compatible, inexpensive and often recyclable.^41^ Diacetoxyiodobenzene was reported to selectively cleave a series of dipeptides (5) containing an N-terminal tyrosine under mild conditions (Fig. 1b).^42^ The bond scission occurred between the C^α^ and C^β^ of the Tyr residue in intermediate 6, generating 4-methylenecyclohexadienone (7) which was then converted to the N-terminal fragment 4-(methoxymethyl)phenol (8) in presence of MeOH. Concomitantly, an intact C-terminal amino acid 9 was obtained from an acyl cyanide species 11 after hydrolysis and decarboxylation (Fig. 1b). Due to the limited substrate scope for N-terminal tyrosyl peptides, no additional studies or applications of this reaction have been reported in the literature.

**Fig. 2:**
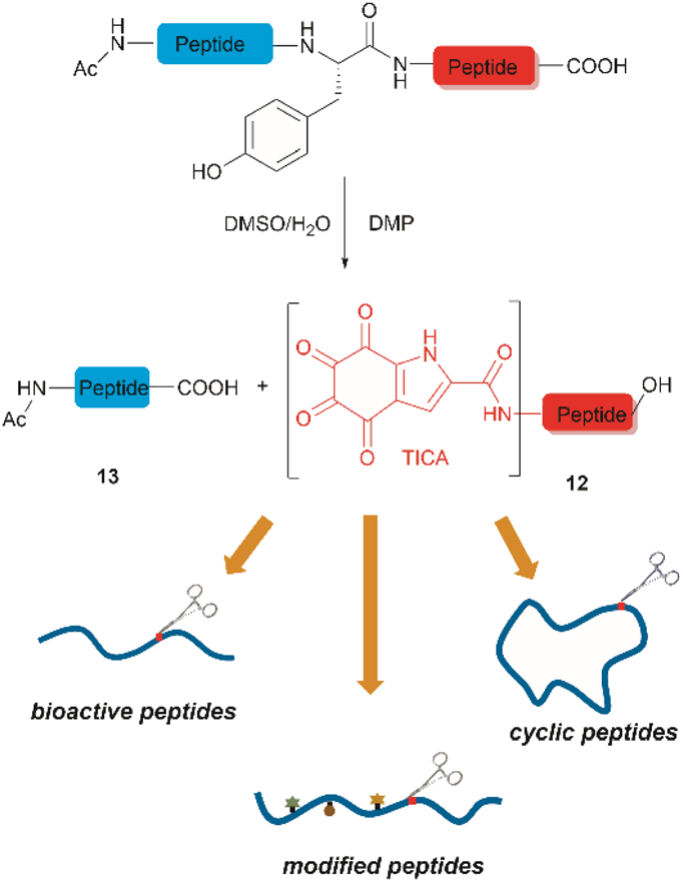
The DMP-mediated oxidative cleavage at peptide bond at Tyr sites.

Inspired by a serendipitous finding in the synthesis of naturally occurring cyclic peptide callyaerin A,^43^ another hypervalent iodine compound, Dess–Martin periodinane (DMP), was recently identified by our group as a highly selective and robust peptide-bond cleaving reagent targeting Tyr.^44^ The scission of the N-terminal amide bond of Tyr by DMP was achieved in a mixture of DMSO and PBS buffer (pH 7.0) at 40 °C, generating a C-terminal peptide fragment 12 bearing the unprecedented hyperoxidized tyrosine motif, 4,5,6,7-tetraoxo-1*H*-indole-2-carboxamide (TICA), along with an unmodified N-terminal peptide fragment 13 (Fig. 2). Compared to the previous cleavage methods targeting Tyr, this DMP-mediated hyperoxidative cleavage approach exhibited superior performance due to several advantages. Firstly, it demonstrated high selectivity for Tyr without impacting other oxidation-sensitive residues like Trp, Ser, and Thr. Secondly, it showed high functional group tolerance of proteinogenic and several unnatural amino acids and led to no modification of other amino acid residues, except for Cys. Thirdly, it achieved high conversion rates exceeding 80% for most tested substrates. Using this method, we successfully cleaved a series of biorelevant oligopeptides ranging from 10–30 residues. However, it was later discovered that the presence of a free peptide N-terminal amine hampers the amide bond cleavage reaction at Tyr. Therefore, an additional acetylation of the N-terminal amine is required before performing the DMP-mediated cleavage. Moreover, we achieved the cleavage of three naturally occurring cyclic peptides, including one depsipeptide and one lipopeptide, using DMP. The generated linearized peptides from the cleavage reaction significantly simplify cyclic peptide sequencing by MS/MS, providing a robust tool to facilitate rapid sequence determination of diverse cyclic peptides containing tyrosine. It is worth noting that the generated TICA moiety derived from either DMP-mediated peptide cleavage or direct oxidation of a peptidyl N-terminal Tyr features four contiguous ketone functionalities. The highly electrophilic nature of TICA renders it a reactive target for the selective bioconjugation or synthetic manipulation of peptides containing this moiety.

### 2.2 Electrochemical methods

**Fig. 3:**
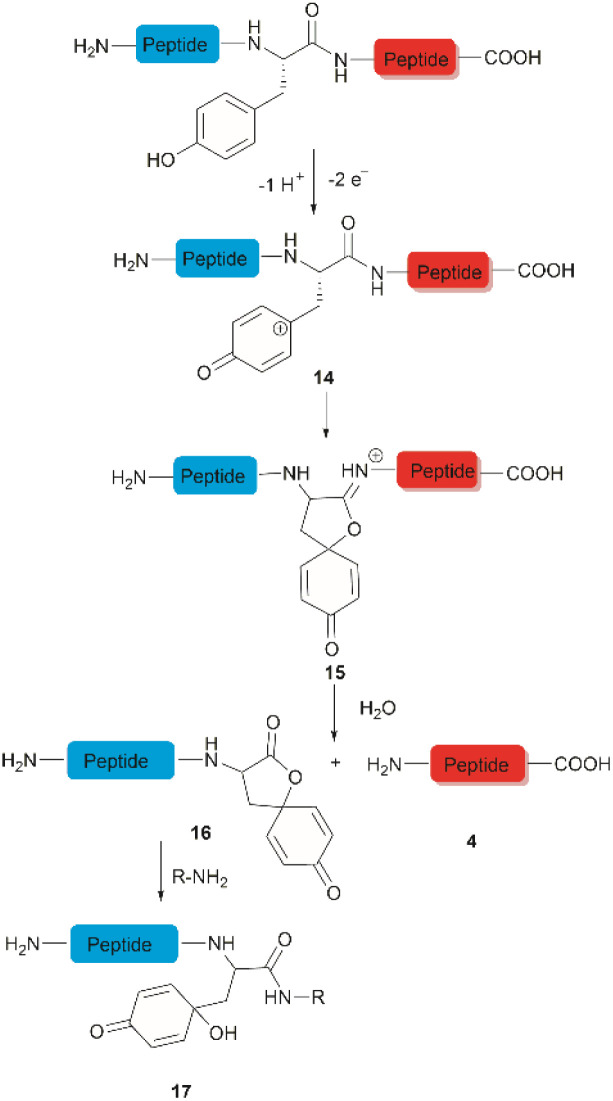
Tyr-selective cleavage of peptide bond *via* electrochemical approach and selective labelling of the resulting N-terminal peptide fragment 16 by aminolysis.

The selective hydrolysis of peptides and proteins at Tyr sites can be also accomplished *via* electrochemical methods. Bruins *et al.* have revealed that electrochemical oxidation of Tyr and Trp within peptides leads to specific cleavage of the amide bond at their C-terminal side.^32,45,46^ Upon electrochemical oxidation of the phenol group of Tyr, a phenoxonium group 14 is generated. This intermediate can then undergo lactonization with the carbonyl group of the C-terminal amide to give intermediate 15, which upon hydrolysis yields an N-terminal peptide fragment containing a spirodienone-lactone unit 16 and an unmodified C-terminal peptide 4 (Fig. 3). Achieving site-selectivity of Tyr over Trp is challenging due to their similar oxidation potential in acidic solutions, which are the optimal conditions to achieve the cleavage reaction.^47^ Met and Cys were also oxidized during this process, but disulfide bonds remained intact.

A wide variety of substrates, ranging from tripeptides to proteins, have been successfully cleaved at Tyr and Trp sites in electrochemical cells with low to moderate yield.^32,46^ The most remarkable feature of this cleavage method is its compatibility with MS and LC-MS analysis. The electrochemical cell can be directly coupled to a mass spectrometer, thereby allowing fast and real-time analysis of the complex protein digest without the need for extra sample preparation. However, the electrochemical oxidation of proteins and peptides suffered from inherent low yields as a mixture of non-cleavable oxidation products was generated simultaneously during the cleavage. It is also interesting to note that the generated spirolactone moiety of the cleaved N-terminal fragment 16 is susceptible to nucleophilic attack. Aminolysis of the spirolactone with several amine-containing tags, such as biotin and fluorescent dyes, would afford compound 17, enabling selective labelling and enrichment of electrochemically cleaved peptides (Fig. 3).^31^

### 2.3 Enzymatic methods

**Fig. 4:**
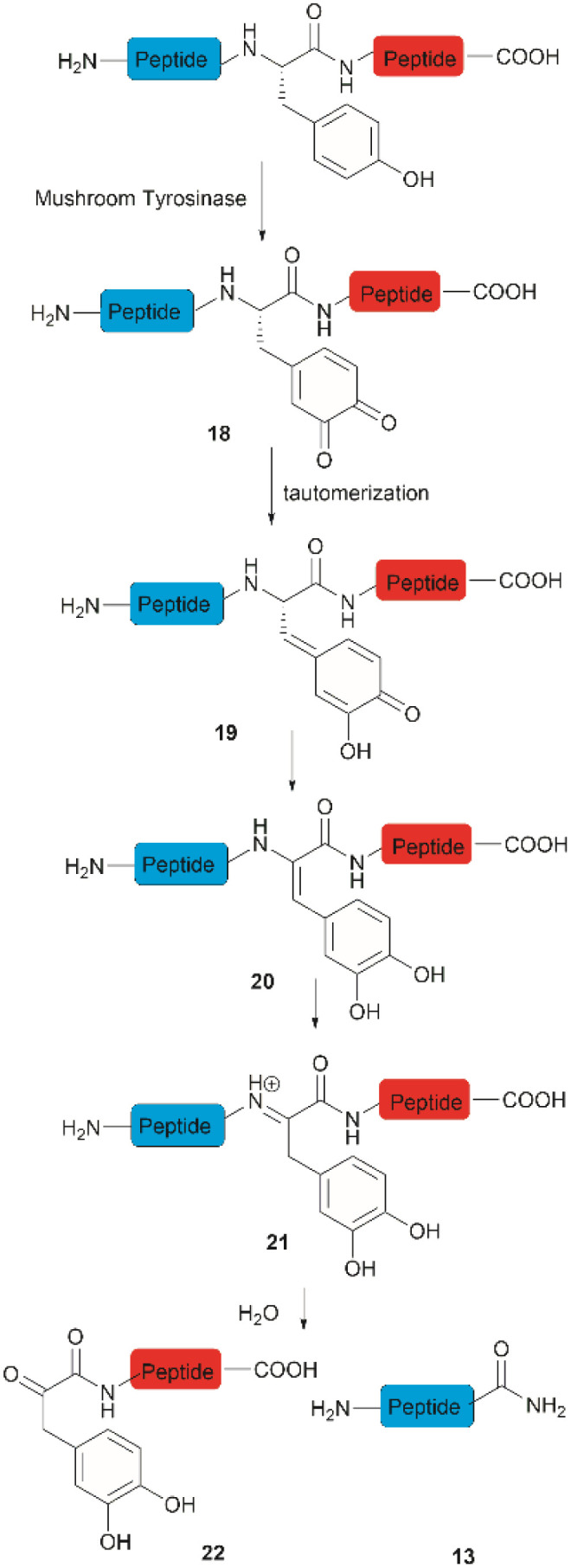
The enzymatic cleavage of proteins at Tyr sites.

Accounts of the enzymatic hydrolysis of the peptide bond adjacent to Tyr are exceedingly scarce. Mushroom tyrosinase is the only enzyme that has demonstrated the capability to catalyze this transformation. As reported by Long and Hedstrom, an unstructured hemagglutinin tag YPYDVPDYA attached to *Escherichia coli* dihydrofolate reductase was selectively cleaved by mushroom tyrosinase at the N-terminal amide of Tyr *via* an *ortho*-quinone intermediate 18 (Fig. 4).^34^ It was proposed that this quinone species could undergo a series of tautomerizations, affording an acyl enamine species 20 with concomitant rearomatization of the arene ring. The acyl enamine 20 is labile to hydrolysis in aqueous conditions, leading to the formation of a C-terminal α-keto amide 22 and an N-terminal peptide amide 13. However, the presence of exogenous nucleophiles and a high protein concentration disfavors this process owing to interception of the generated *ortho*-quinone and potential protein cross-linking. Furthermore, the growing significance of post-translationally modified Tyr residues in normal and diseased biological processes has prompted the development of proteases that selectively cleave these residues, such as phosphotyrosine, *O*-sulfated tyrosine, and 3-nitrotyrosine. One engineered bacterial protease subtilisin BPN variant targeting phosphotyrosine^48^ and two *E. coli* outer membrane protease (OmpT) variants aiming for *O*-sulfated tyrosine^49^ and 3-nitrotyrosine^50^ were identified to hydrolyze peptides at the C-terminal amide of the modified Tyr residues with high specificity.

## 3 Selective functionalization and conjugation at Tyr residues in proteins/peptides

This section will cover recent advancements from the last three years in the functionalization of tyrosine residues and conjugation, highlighting developments not covered in earlier reviews. Functionalization will cover the addition of functionalities to Tyr residues. These functionalities do not necessarily have a specific bioactivity or function by themselves, a point that differentiates functionalization from conjugation but are added to Tyr to fine-tune or optimize protein/peptide properties (*e.g.*, stability, pharmacokinetics, *etc.*) for diversification or post-modification purposes, topics of particular interest in medicinal chemistry.

### 3.1 Tyr functionalization

The incorporation of modified amino acid residues into a peptide or protein is of interest as it provides access to modified natural biologics that could be used in applied areas such as proteomics, diagnostics, asymmetric syntheses, and drug delivery.^51^

#### 3.1.1. Chemical functionalization/modification of Tyr

**Fig. 5:**
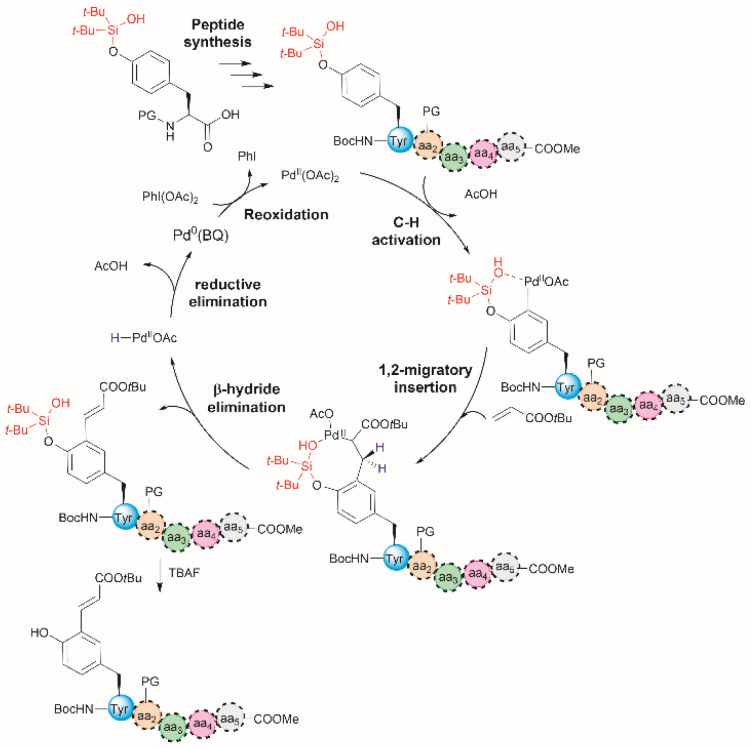
Proposed mechanism for the Pd-catalyzed *ortho*-olefination of peptides. Reaction conditions: protected peptide (0.2 mmol, 1.0 equiv.), acrylate *tert*-butyl ester (0.8 mmol, 4.0 equiv.), Pd(OAc)_2_ (0.02 mmol, 0.1 equiv.), BQ (0.04 mmol, 0.2 equiv.), PhI(OAc)_2_ (0.6 mmol, 3.0 equiv.), and Li_3_PO_4_ (0.4 mmol, 2.0 equiv.) in dichloroethane (DCE) (2.0 mL) at 90 °C for 24 h. PG = protecting group.

In 2020, a late-stage Pd-catalyzed *ortho*-olefination of *O*-silanol-protected Tyr was reported by Hu *et al.*^52^ This method involves introducing a silanol unit bearing a dual functionality as a hydroxyl-protecting group and a site-directing group for olefination. The reaction additives required to carry out this reaction comprised the oxidant PhI(OAc)_2_, benzoquinone (BQ) and a base. The role of BQ is believed to prevent the aggregation of the Pd(0) species by forming a Pd(0)-BQ complex and to facilitate oxidation to Pd(ii).^53,54^ The proposed reaction mechanism involves consecutive C–H activation, transmetalation, reductive elimination, and catalyst re-oxidation steps (Fig. 5).^55–57^ An important limitation of the method is that it requires nucleophilic coordinating functional groups present at the terminal ends and residues' side chains to be protected as they interfere with the catalyst-driven olefination. This protocol is well-suited for solid-phase peptide synthesis. For peptides containing Tyr in the N- or C-terminus, the *ortho*-functionalization method afforded moderate to good yields in the case of dipeptides (66–33%) but yields generally decreased with an increase in the length of the peptide (<23% for pentapeptides). This was attributed to the complexation of Pd(ii) by the protected peptide chain. Aside from the poor reaction yields for longer peptides, the *ortho*-olefination comprises poor atom economy. One advantage of the method is that the removal of the silanol group is easily accomplished in presence of tetrabutylammonium fluoride (TBAF). It is important to note that Hu *et al.*^52^ reported that for a dipeptide containing Phe at the C-terminus, the Phe residue remained unchanged when the peptide was subjected to the reaction conditions, thereby highlighting the chemoselectivity of the reaction.

**Fig. 6:**
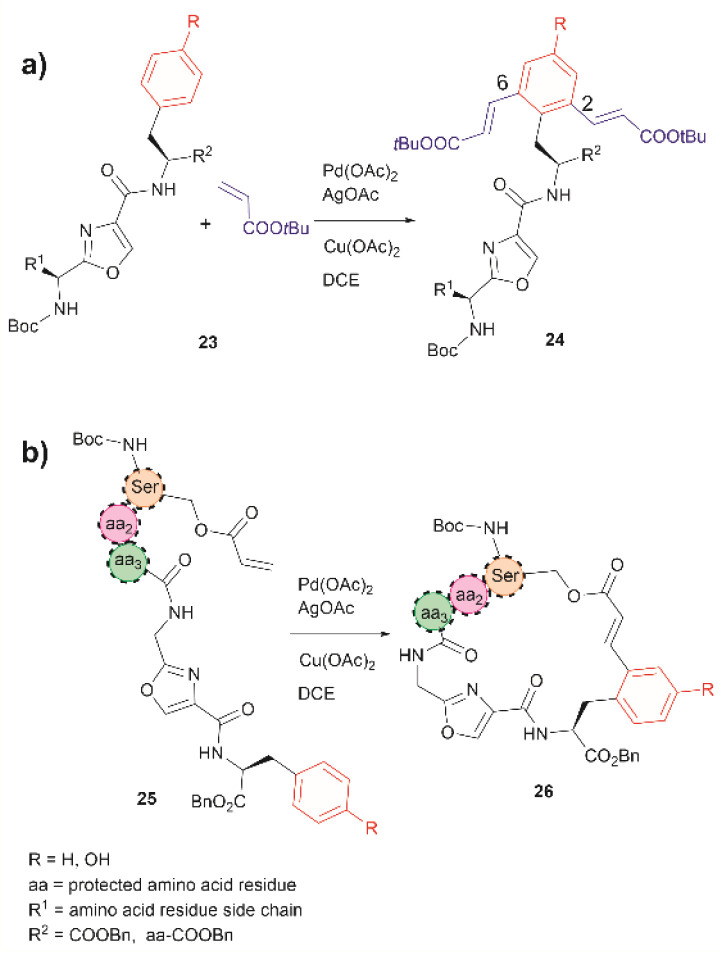
(a) Intra- and (b) inter-molecular Pd catalyzed C–H olefination of peptides containing an internal oxazole unit. Reaction conditions: peptide (0.10 mmol), acrylate *tert*-butyl ester (4.0 equiv.), Pd catalyst (10 mol%), AgOAc (4.0 equiv.), Cu(OAc)_2_ (4.0 equiv.), in DCE (2.0 mL) at 100 °C for 24 h.

In 2021, Liu *et al.* reported that an oxazole unit within a peptide sequence acts as an internal directing group for the late stage Pd catalyzed C–H olefination of peptides containing Phe, Tyr or Trp residues located contiguously at the C-terminal of the oxazole (23 in Fig. 6a).^58^ This process is of practical value for the functionalization/modification of natural peptides containing oxazoles^59^ or other heterocyclic units that can bind the catalyst. However, protection of the N- and C- terminal groups and residues' side chain coordinating groups is required, thus limiting the application to synthetic oxazole peptide analogues. The oxazole-directed C–H olefination generated the 2,6-disubstituted product 24 for Phe and Tyr-containing di- and tripeptides in moderate to good yields (44–80%). Furthermore, Liu *et al.* also demonstrated how this oxazole-enabled C–H olefination protocol can be used to cyclize peptide 25 to 26 (Fig. 6b). Importantly, the application of the oxazole-directed olefination to longer peptides is yet to be reported.

**Fig. 7:**
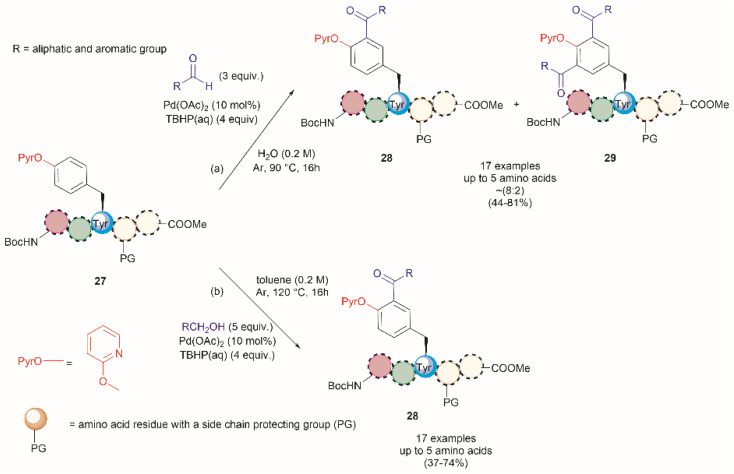
Pd catalyzed C–H acylation of Tyr containing peptides with (a) aldehydes and (b) alcohols in aqueous media using a 2-pyridil ether unit as a directing group.

A common feature among the Pd-catalyzed C–H olefination reactions discussed above is the use of organic solvents, which is partly due to the high hydrophobicity of the protected peptides. In 2020, San Segundo *et al.* reported the Pd-catalyzed *ortho*-acylation of Tyr-containing peptides 27 in aqueous media in the presence of aldehydes using a 2-pyridyl ether unit (OPyr) as a directing group (path a, Fig. 7).^60^ Aldehydes were the acylating agents and *tert*-butylhydroperoxide (TBHP) was the oxidant. Under optimized conditions a protected dipeptide (Boc-NH-(OPyr)Tyr-Leu-CO_2_Me) formed mono- and di-acylated product 28 and 29 with a variety of aromatic aldehydes in an up to 8 : 2 ratio affording moderate to good yields (44–81%) of final isolated products. Interestingly, only moderate yields of the mono-acylated product were observed in the reaction with heptanal or cyclohexanecarboxaldehyde which was attributed to the lower oxidation tendency of aliphatic aldehydes relative to their aromatic counterparts. A slight increase in yield (up to 57%) resulted using an excess of the aliphatic aldehyde in chlorobenzene. Similarly, the reaction of aldehydes of heterocyclic aromatic compounds used in excess produced the mono-acylated compounds when toluene was used as a solvent with the reaction carried out at 100 °C. These results underscore the crucial role of the solvent in controlling the reaction selectivity. However, while a Pd^II^/Pd^IV^ reaction mechanism has been proposed for similar procedures,^61^ a full understanding of the effects of each reaction component is still required. Importantly, the reaction was successfully applied to the late-stage acylation of peptides as long as six amino acids, regardless of the position of the (OPyr)Tyr unit, with no interference of Gln, Asn, Ser and Tyr, having unprotected side chains or protected Lys. However, as expected, residues such as His, Trp, Arg, Cys and Met were not tolerated.

Additional applications of the Pd-catalyzed acylation reaction described above include: (1) the preparation of asymmetrical diacylated peptides, and (2) the linkage of two peptide units granted that one has an aldehyde moiety installed within the peptide sequence. However, it should be noted that the proof of principle for these reactions, as reported by San Segundo *et al.*,^60^ is limited to hydrophobic protected peptide chains due to the use of toluene as the solvent. Of further concern is the removal of the OPyr group, as conditions for its efficient excision are yet to be reported, thus limiting the applicability of the method. Segundo *et al.* were able to remove the OPyr directing group following a Ni-catalyzed borylation protocol, but poor reaction yields were reported (41%).

In a further attempt to improve the Pd-catalyzed acylation of (OPyr)Tyr-containing peptides, the same research group reported the use of alcohols as acylating reagents instead of aldehydes.^62^ This research also aimed to demonstrate the feasibility of using EtOH as a cheap and environmentally friendly acylation reagent. To this end, they first showed that a simple model dipeptide Boc-NH-(OPyr)Tyr-Leu-CO_2_Me could exclusively produce the *ortho*-, mono-acetylated Tyr product in the presence of EtOH using Pd(OAc)_2_ as the catalyst, an aqueous solution of TBHP as the oxidant, and toluene as the solvent at 120 °C. Importantly, it was also shown that the protocol was not limited to the use of EtOH as a coupling partner since monoacylated peptides were also obtained in the presence of other aliphatic and benzyl alcohols in moderate to good yields (37–74%). The same protocol was also successfully applied to peptides containing up to 5 residues (path b, Fig. 7), but the introduction of protecting groups for side chain functional groups that can be oxidized (such as Lys, Ser, Tyr, Asp, Glu, and Arg) was required, in addition to the N- and C-terminal groups. While peptides containing His and Trp were not tested in this work, one can presume that these residues will also be acetylated under the reported conditions. The location of the OPyrTyr residue at different positions of the peptide did not affect the Pd-catalyzed acylation reaction.

**Fig. 8:**
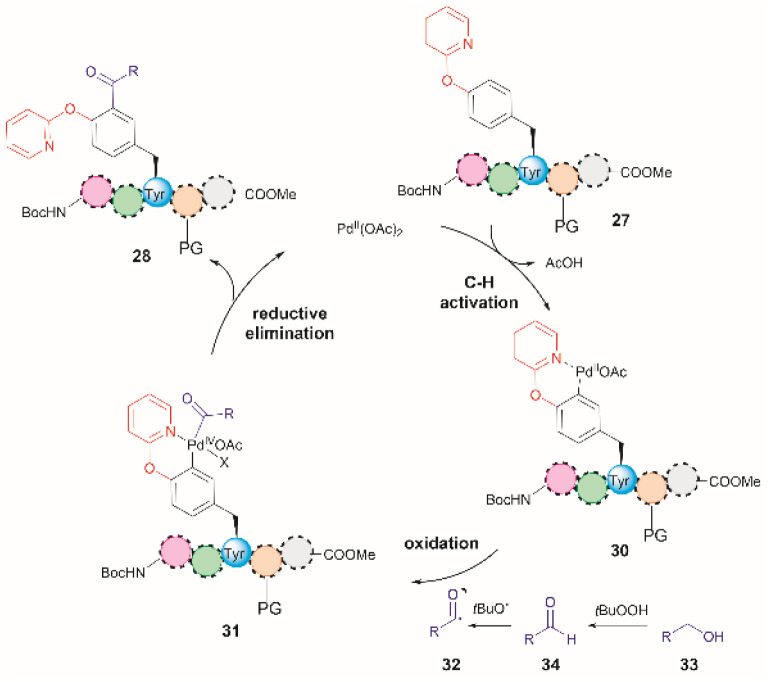
Proposed mechanism for the Pd-catalyzed *ortho*-acylation of peptides.

The authors of this work also performed a few experiments to gain insights into the reaction mechanism of the directed Pd-catalyzed *ortho*-acylation. These studies led them to propose a Pd^II^/Pd^IV^ process *via* the formation of an acyl radical 32, presumably derived from the oxidation of the alcohol 33 to the corresponding aldehyde 34 (Fig. 8). Interestingly, when acetaldehyde was used instead of EtOH in toluene at 120 °C a mixture of mono- and diacetylated products was obtained. Therefore, it is currently unclear why the selectivity for mono-acetylation occurs when alcohols are utilized instead of their corresponding aldehyde counterparts.

**Fig. 9:**
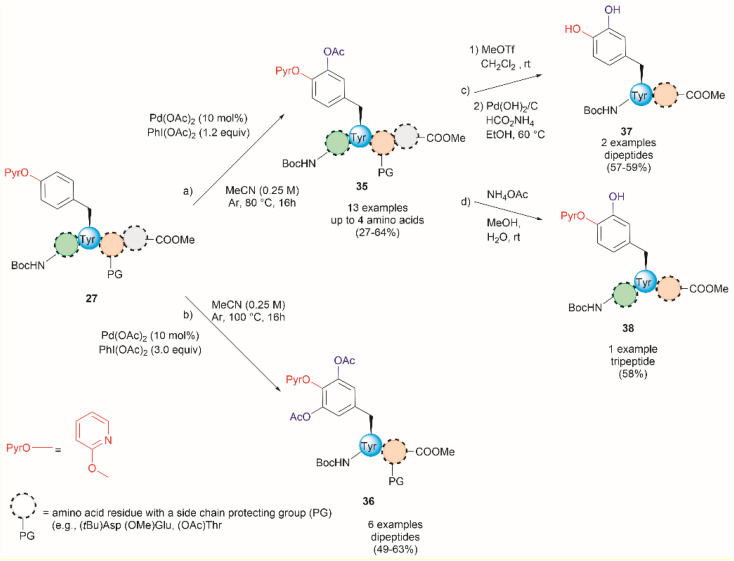
Pd-catalyzed (a) monoacetoxylation and (b) diacetoxylation of peptides. (c) Directing group removal with concomitant hydrolysis of the acetyl group. (d) Chemoselective hydrolysis of the acetyl group.

An extension of the previous work is the Pd-catalyzed *ortho*-acetoxylation of *O*-pyridinium protected Tyr peptides.^63^ This process was successfully accomplished using Pd(OAc)_2_ as the catalyst, and PhI(OAc)_2_ as the oxidant in acetonitrile. Changes in the equivalents of oxidant and temperature allowed for the controlled formation of either mono- or diacetoxylated products 35 or 36 (Fig. 9). Thus, the mono-acetoxylated compound 35 was primarily obtained in presence of 1.2 equiv. of PhI(OAc)_2_ at 80 °C and the diacetoxylated product 36 was exclusively obtained with 3.0 equiv. of oxidant at 100 °C. The protocol was successfully applied for the monoacetoxylation of peptides up to 4 residues long (path a, Fig. 9) and diacetoxylation of dipeptides (path b, Fig. 9) in moderate yields (27–64%) regardless of the location of the (PyrO)Tyr residue within the peptide chain. However, when preparing the monoacetoxylated peptides 35, it was necessary to increase the amount of oxidant and temperature with an increase in the number of residues to reach full conversion. As before, it is important to note that the post-acetylation can only be performed in fully protected peptides, but Phe is tolerated. Furthermore, an improved process for the removal of the PyrO group, comprising consecutive methylation and hydrogenation steps (path c, Fig. 9), was used in this work, resulting in the corresponding l-DOPA peptidomimetic compound 37 in moderate yields (57–59%). Selective hydrolysis of the acetyl group without removal of the PyrO directing group was also carried out with ammonium acetate (path d, Fig. 9). However, the poor overall yields for the final functionalized unprotected peptides and the pending application of the method to longer peptides are some evident limitations of this protocol.

**Fig. 10:**
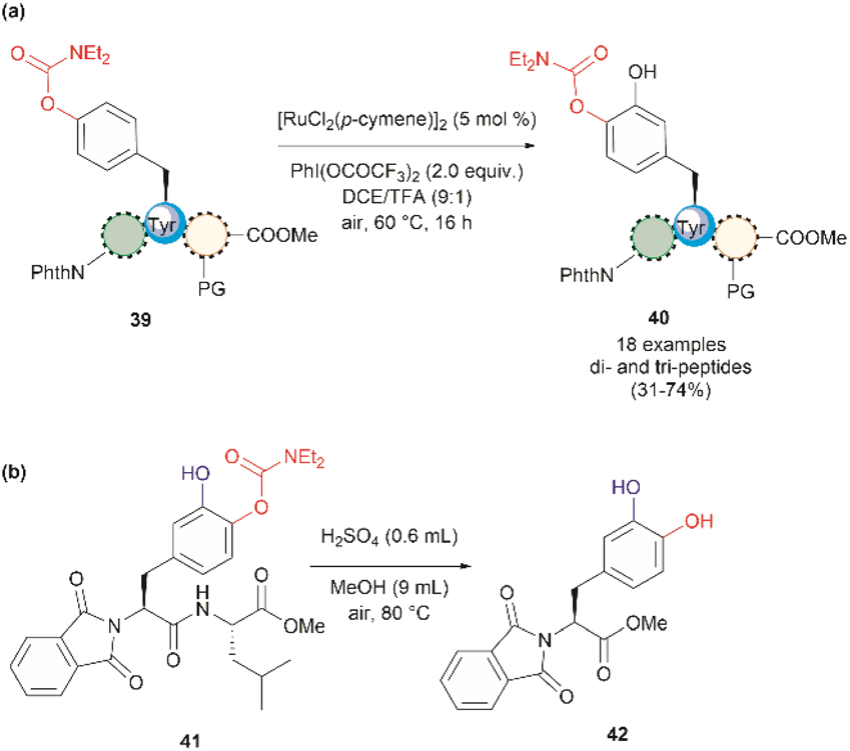
(a) Ru-catalyzed hydroxylation of di- and tri-peptides (the location of the Tyr residue varies within the peptide sequence), (b) cleavage of the carbamate directing group.

In 2022, Andrade-Sampedro *et al.* also reported a Ru-catalyzed late-stage hydroxylation of tyrosine, presenting an alternative approach for incorporating l-DOPA into peptides.^64^ This reaction comprised the use of a Ru catalyst and PhI(OCOCF_3_)_2_ as the oxidant in dichloroethane with an excess of trifluoroacetic acid (TFA). A carbamate was installed in the hydroxyl group of Tyr, which functioned as a directing group during the hydroxylation process. The process implies the *ortho*-trifluoroacetoxylation of Tyr, presumably *via* a Ru^II^/Ru^IV^ mechanism, which leads to the hydroxylation upon hydrolysis of the trifluoroacetyl group. It is important to note that the presence of TFA restricts the use of acid-labile protecting groups during the catalytic hydroxylation. In this work, the authors used phthalimides (Phth) as the amino protecting group and methyl esters as the carboxylic acid protecting group. This poses a drawback to the method given the scarcity of gentle and near-neutral deprotection procedures for these groups. However, the reaction is more robust than the Pd-catalyzed acetoxylation described above since it is air tolerant and can be conducted at moderate temperatures (60 °C).^64^ While the reaction was successfully applied to the hydroxylation of di- and tri-peptides 39 (Fig. 10a), with only the mono-hydroxylated products 40 being isolated, the procedure did not work with longer peptides, presumably due to the coordination of Ru by the amide bonds. Furthermore, a more important restriction for the practical application of the methodology in the preparation of l-DOPA-containing peptides was that peptide cleavage occurred under the conditions required for the removal of the carbamate group (H_2_SO_4_ in MeOH at 80 °C, see Fig. 10b). Therefore, additional research is required to address these identified limitations.

**Fig. 11:**
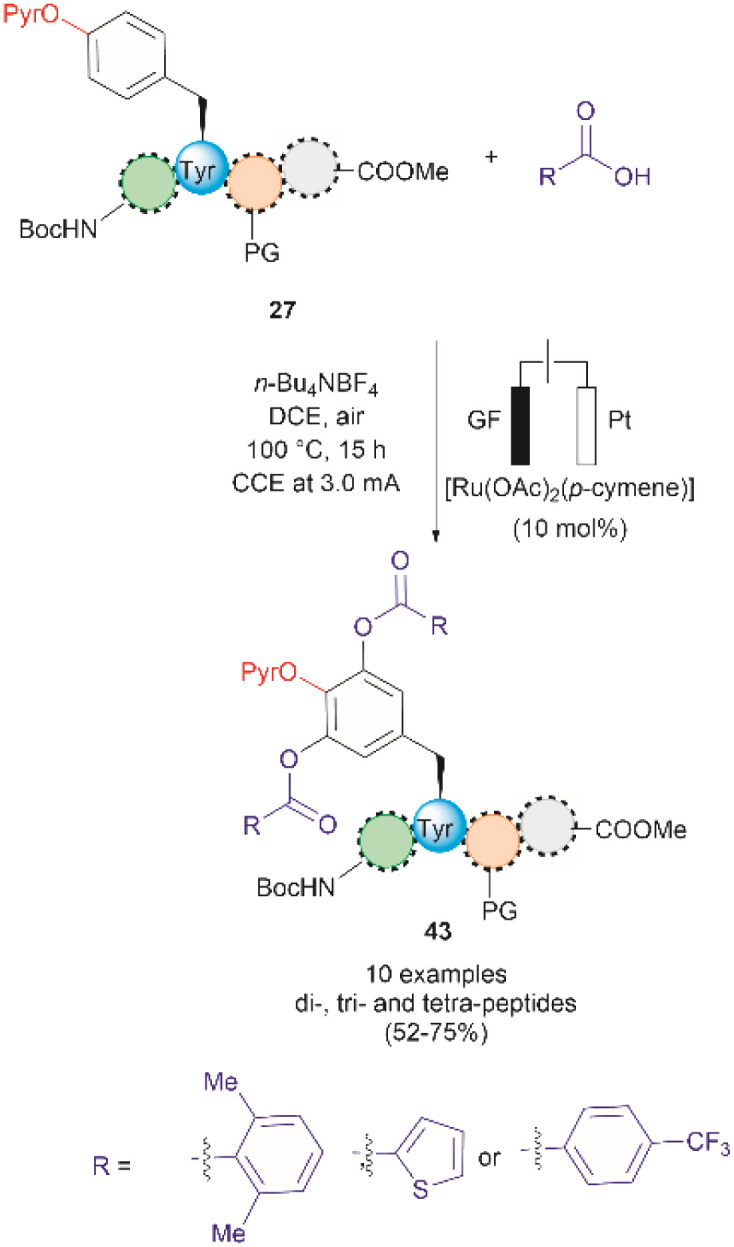
Ru-electrocatalytic *ortho*-acyloxylation of (OPyr)Tyr containing peptides *via* electrooxidation.

A recent report by Hou *et al.* in 2022 presented an advancement in the Ru-catalyzed acyloxylation of Tyr (Fig. 11).^65^ The process utilizes an electrooxidation process instead of using stoichiometric amounts of chemical oxidants, leading to mild reaction conditions. Additionally, a PyrO directing group was employed. Notably, Andrade-Sampedro *et al.* also investigated the PyrO directing group under the reaction conditions used in the Ru-catalyzed hydroxylation of Tyr, but no product was observed. This could be attributed to protonation of the pyridine nitrogen in the highly acidic reaction media, thereby impeding the coordination of Pyr with Ru.^64^ The electrocatalytic process reported by Hou *et al.* was performed in an undivided electrochemical cell comprised of a graphite felt (GF) anode and a platinum plate (Pt) cathode under constant current. Optimized conditions included the use of [Ru(OAc)_2_(*p*-cymene)] as the catalyst and *n*-Bu_4_NBF_4_ as the electrolyte in dichloroethane as the solvent (Fig. 11). Moreover, the reaction also proceeded at reasonable conversion yields when an arene-ligand free catalyst (*e.g.*, [Ru_2_(OAc)_4_Cl] or [RuCl_3_·3H_2_O]) was employed, indicating that the *p*-cymene unit is not participating during the catalytic process. The reaction was robust and could be performed in air, tolerating a wide range of aromatic and aliphatic carboxylic acids when reacted with *ortho*-monosubstituted (OPyr)phenol substrates. Furthermore, the acyloxylation was successfully applied to the late-stage modification of Tyr in peptides up to 4 amino acids long, selectively yielding the *ortho* diacyloxylated product 43 in most cases with few instances where a mixture of mono- and di-acetylated Tyr peptide were also observed. The removal of the Pyr group was accomplished as indicated above using a methylation/hydrogenation protocol (path c, Fig. 9).

**Fig. 12:**
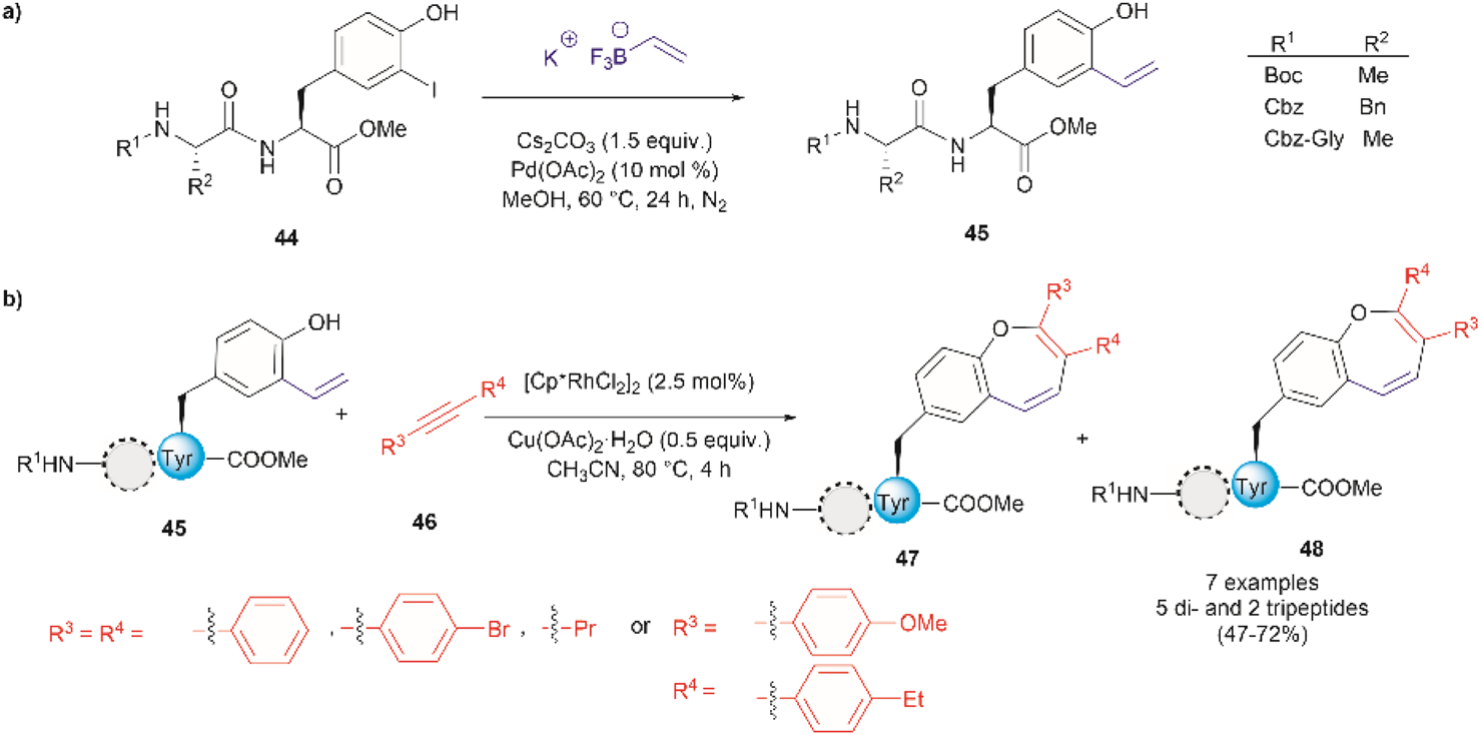
(a) Suzuki–Miyaura cross coupling reaction for the preparation of *ortho*-vinylated Tyr peptides. (b) Rh-catalyzed annulation of *ortho*-vinylated Tyr with alkynes to give functionalized oxepine peptides.

Thus far, we have reviewed transition metal-mediated tyrosine functionalization in peptides. Importantly, it is essential to provide examples of Tyr-containing peptide functionalization combined with further modifications, such as the recent study by Kharat *et al.*, who demonstrated a Rh-catalyzed annulation of *ortho*-vinylated Tyr with alkynes, resulting in functionalized oxepine dipeptides and tripeptides.^51^ The benzo[*b*]oxepine heterocyclic is found in several natural products and has constituted an important scaffold for the development of bioactive compounds, hence synthesizing peptides containing the oxepine heterocyclic system is highly desired. Peptide 44 was first prepared *via* well-established liquid-phase peptide synthesis protocols. *Ortho* vinylation of Tyr in these peptides was then carried out by a Suzuki–Miyaura cross-coupling reaction (Fig. 12a).^66^ Optimized conditions to achieve the [5 + 2] annulation between the peptide 45 containing an *ortho*-vinylated Tyr residue and alkyne 46, comprised the [Cp*RhCl_2_]_2_/Cu(OAc)_2_·H_2_O catalytic system in acetonitrile (CH_3_CN) at 80 °C. The reaction of alkynes and di- and tri-peptides furnished the expected annulated products in fair to good yields (47–72%) (Fig. 12b). The reaction of an asymmetrical alkyne and a dipeptide yielded a mixture of two regioisomeric compounds 47 and 48. Further attempts to apply this method to longer peptides are still required.

**Fig. 13:**
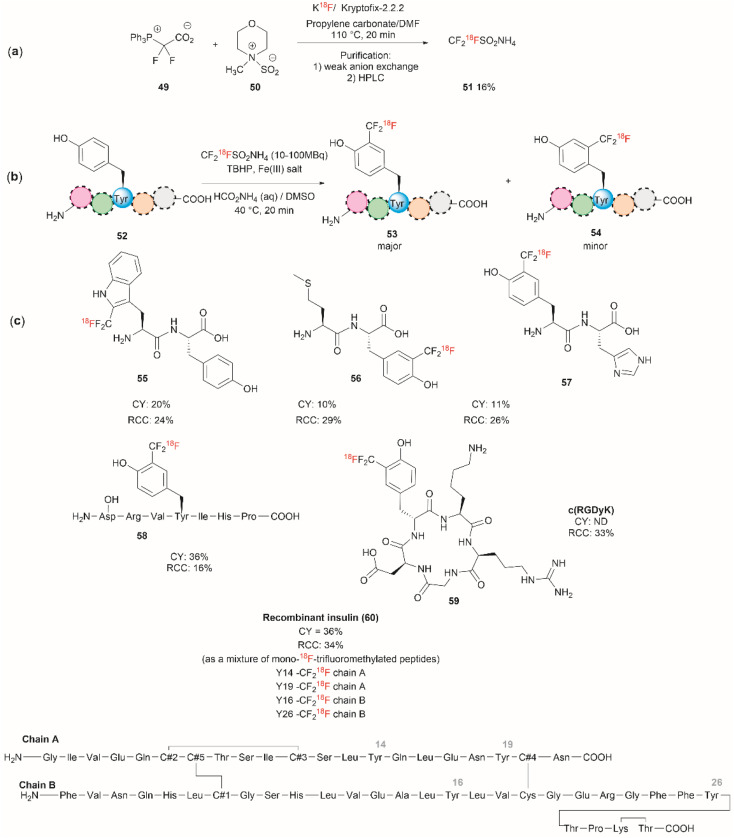
(a) Chemical synthesis of ammonium ^18^F-trifluoromethanesulfinate. (b) General conditions for the ^18^F-trifluoromethylation of Tyr containing peptides. (c) ^18^F-trifluoromethylated peptides.

Although it has been part of the discussion of another review, we will also expound on the one-step ^18^F trifluoromethylation of Tyr-containing peptides reported by Kee *et al.*^67^ Even though this reaction is not specific for Tyr, since Trp could also be modified under the same reported conditions, it is of relevance as it allows the fast and relatively easy preparation of ^18^F radiolabelled proteins/peptides. ^18^F is a short-lived radioisotope (*t*_1/2_ = 109.7 min) widely used in positron emission tomography (PET). Thus, ^18^F functionalized Tyr biomolecules can be used to monitor the pharmacokinetics and biodistribution of therapeutics, as well as many *in vivo* biological processes. Inspired by the work of Krska *et al.*,^68^ who used zinc trifluoromethanesulfinate and an oxidant or photoredox catalyst activator to perform the trifluoromethylation of tyrosine-containing peptides, Kee *et al.*^67^ started with 2,2-difluoro-2-(triphenylphosphonio)acetate (49) as a source of difluorocarbene,^69^ and *N*-methylmorpholine·SO_2_ (50) as a surrogate SO_2_ source and K^18^F to synthesize ammonium ^18^F-trifluoromethanesulfinate (51) (Fig. 13a). With 51 in hand, the authors then optimized the reaction conditions for the ^18^F-trifluoromethylation of peptides containing Tyr and/or Trp. The reaction was performed in the presence of TBHP as an oxidant, an Fe(iii) salt in aqueous ammonium formate and DMSO as co-solvent. The C3 substituted product 53 appeared to be the dominant species, along with a small amount of C2 substituted product 54 (Fig. 13b). The process was applied to the synthesis of a series of dipeptides (55–57), the angiotensin I/II (1–7) linear heptapeptide (58), the α_V_β_3_ integrin-binding RGD cyclic pentapeptide c(RGDyK) (59), and recombinant insulin (60) (Fig. 13c). The chemical reaction yields (CY) for the trifluoromethyated peptides were poor (10–36%). Moreover, the protocol for peptide radiolabeling had to be further customized to fit the operational time demands of ^18^F-radiotracking. Thus, final ^18^F-trifluoromethylated peptides with radiochemical conversions (RCC) between 16% to 34%, calculated as a percentage of all radioactive compounds detected by radio-HPLC, were obtained. The protocol was slightly modified for its introduction into a fully automated radiosynthesis system in which a model peptide total synthesis time (from K^18^F to CF_2_^18^F-peptide) was around 133 min, which is within the processing and formulation times reported for other systems.^70^

Salient features of the ^18^F-trifluoromethylation reaction are: (1) when both Tyr and Trp are present in a peptide ^18^F-trifluoromethylation occurs exclusively in the Trp residue; (2) the reaction is regioselective, as the C3 substituted product 53 was the major product obtained for Tyr, however; (3) the reaction is not regiospecific, since a separable regioisomer 54 at C2 was also isolated; and (4) the reaction is tolerant to the presence of most unprotected amino acid residues, except to Cys, which gets oxidized to cystine.

**Fig. 14:**
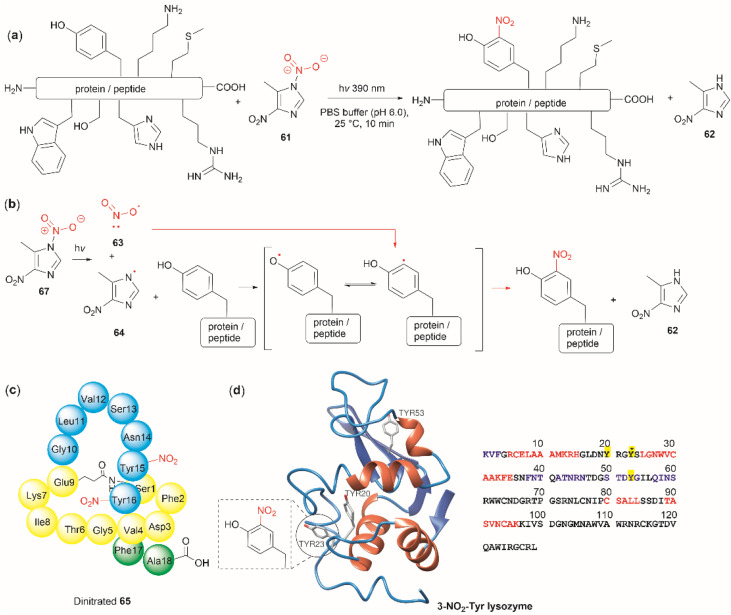
(a) General reaction and (b) mechanism of the light-controlled chemoselective 3-nitration of Tyr of proteins and peptides. (c) Structure and amino acid sequence of nitrated lasso peptide caulonodin IV (65) and (d) lysozyme (PDB ID: 1LYZ) with its primary amino acid sequence with Tyr residues highlighted in yellow.

The nitration of Tyr is a crucial oxidative post-translational modification (PTM) of proteins involved in numerous physiological processes and diseases, such as cardiovascular and neurodegenerative diseases, inflammation, and aging.^71,72^ Additionally, bioactive nitro-Tyr-containing peptides are also found in nature.^73,74^ Despite its biological importance, efficient and selective methods to introduce the nitro group to tyrosine residues in proteins are lacking. Recently Long *et al.* reported the light-controlled chemoselective 3-nitration of Tyr in proteins and peptides using 5-methyl-1,4-dinitroimidazole (DNIm, 61).^75^ The nitration was carried out in an aqueous buffer under irradiation at 390 nm (Fig. 14a) with concomitant release of 5-methyl-4-dinitroimidazole (62). According to the authors, the light-induced nitration with DNIm (61) proceeds *via* a radical reaction mechanism, where light initially cleaves the N(1)–NO_2_ bond in DNIm, with this being the rate-limiting step. The cleavage yields the nitryl 63 and NIm radical 64. The latter generates a tyrosyl species by removing the hydrogen from the phenol system, which subsequently reacts with the nitro radical to generate the 3-nitrotyrosine (Fig. 14b).

The most salient feature of the DNIm light-induced nitration is its high chemoselectivity towards Tyr, as the side chains of other residues (*e.g.*, Arg, Asp, Glu, His, Lys, Met, Phe, Ser, Thr and Trp) remain intact. However, Cys was oxidized to the corresponding disulfide product. Clear mechanistic evidence of why DNIm is specific to Tyr is lacking, but this could be attributed to a combination of the bond dissociation energy of Tyr and its polarity.^76^ Moreover, based on the similarity in oxidation potential values between Tyr and Trp,^77–79^ one would expect Trp to be also nitrated with this procedure. Thus, further details on the chemoselectivity of the photochemical DNIm-mediated nitration are needed for the future design and development of Tyr selective functionalization reagents. Another aspect to consider in this reaction is the accessibility and stability of DNIm (61). By comparison with similar reagents, it is expected that 61 will be explosive, although reactions in the solution phase pose a lower risk. Furthermore, DNIm (61) in its solid forms was indicated to be a strong irritant and can provoke allergies, hence care must be taken during its manipulation.^80^ While sourcing details of DNIm (61) were not specified by Long *et al.*,^75^ the synthesis of 61 implies the use of harsh conditions^81^ (*e.g.*, nitric acid in glacial acetic acid and acetic anhydride).^82^ Therefore, the development of greener nitration conditions is desirable.

Long *et al.*^75^ demonstrated the robustness of the DNIm chemoselective nitration of Tyr with several peptide substrates (3 to 6 aa long) with excellent conversions (>95%). Of note is the nitration of the more complex lasso peptide caulonodin IV (65). Lasso peptides exhibit a knot topology, whose stability is primarily attributed to the residues positioned above and below the macrolactam peptide ring. In the case of caulonodin IV, Phe17 plays a major role in the stability of the lasso fold.^83^ However, Long *et al.* demonstrated an enhancement in stability against lasso unfolding upon nitration of residues Tyr15-16 (Fig. 14c). This further emphasizes the structural nuances that are determinants of the stability of the knotted structure.

The light-controlled chemoselective 3-nitration of Tyr of several proteins was also demonstrated (lysozyme, RNase A, histone-H2A and α-synuclein). As an illustrative example, we discuss the nitration of lysozyme. Despite having three Tyr residues, nitration of lysozyme resulted in only the mono-nitrated product at Tyr23 (Fig. 14d), even in the presence of excess DNIm (14 equiv. relative to the total protein). This highlighted that residue accessibility is a prerequisite for nitration to occur. Importantly, no other residues were modified during the nitration (*ca.* lysozyme also contains 8 Trp residues and 8 Cys as disulfide residues) further emphasizing the robustness of the method.

Takayama reported the nitration of Tyr in peptides when performing UV-MALDI characterization experiments with a 3,5-dinitrosalicylic acid matrix.^84^ The author suggested that nitrate radicals are formed upon exposure of matrix molecules to UV radiation with subsequent nitration of the Tyr residues of the peptide according to the mechanism proposed by Long *et al.*^75^ (Fig. 14b). Unfortunately, the peptides tested by Takayama did not contain Met, His or Trp, which are residues known to be easily oxidized. Hence, the full assessment of the chemoselectivity of the process is pending. However, for peptides containing Arg, Asp, Cys, Glu, Ser, Thr and Phe and other aliphatic amino acids, nitration was observed exclusively at Tyr. Interestingly, for a nonadecapeptide containing 6 glycine residues several [*a* + 16]^+^ ion fragments were detected. These corresponded to the cleavage of C_α_–C bonds of Gly–Xxx residues and oxidation of the C_α_ of Gly by hydroxyl radicals. This result was surprising as the C_α_–C bond cleavage is rarely observed in MALDI experiments. The formation of hydroxyl radicals was attributed to the 3-NO_2_-Tyr residue as the same cleavage oxidation at Gly sites was not observed in the UV-MALDI spectrum of an unrelated peptide containing Gly but lacking Tyr. More studies are needed to confirm this observation since previous reports indicated that 3-NO_2_-Tyr peptides can generate hydroxyl radicals under photolysis at wavelengths lower than 320 nm while other oxidizing species are formed at higher wavelengths (*e.g.* 355 nm),^85^ which is the excitation wavelength of the standard MALDI lasers. Of further interest is to demonstrate the use of 3,5-dinitrosalicylic acid for the chemoselective nitration of Tyr in the solution phase.

**Fig. 15:**
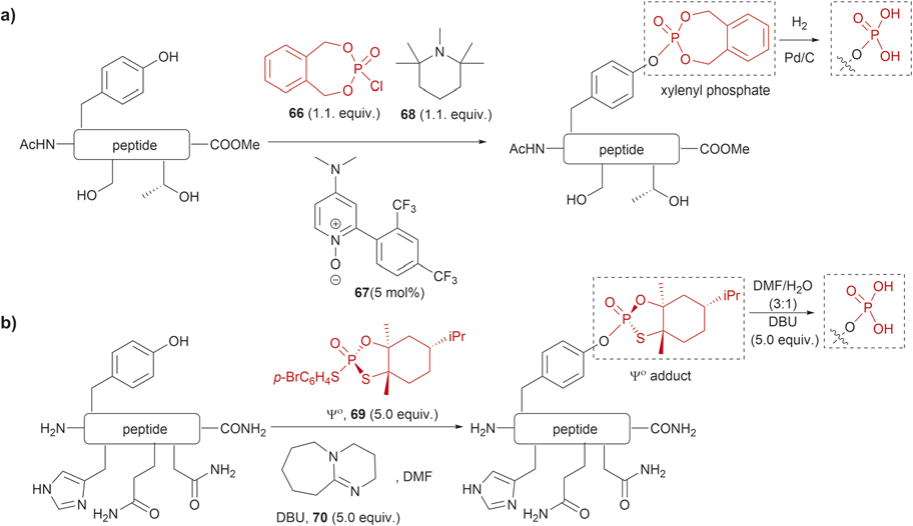
Late-stage phosphorylation of a Tyr-containing peptide with (a) *o*-xylenyl phosphoryl chloride and (b) the P(v) reagent, Ψ^o^.

The phosphorylation of Tyr in proteins is another PTM of importance in many biological processes.^86^ Therefore, the synthesis of peptides/proteins containing phosphotyrosine is of high relevance to understanding the biological role of proteins in signaling pathways of normal and disease states, protein characterization and drug design. The chemical synthesis of phosphotyrosine and phosphonate-based phosphotyrosine analogues has been recently reviewed by Makukhin and Ciulli.^87^ Most synthetic methods focus on the preparation of protected phosphotyrosine analogues as building blocks in solid phase peptide synthesis. However, chemical methods for the late-stage preparation of peptides/proteins containing phosphotyrosine residues are scarce. One of the methods reported in the literature for the site-selective Tyr post-phosphorylation of peptides involved the use of *o*-xylenyl phosphoryl chloride (66), 2-(2,4-bis(trifluoromethyl)phenyl)-4-(dimethylamino)pyridine *N*-oxide (67) as a catalyst and 1,2,2,6,6-pentamethylpiperidine (68) as a base. The phosphate group can be obtained after Pd-catalyzed hydrogenation of xylenyl phosphate (Fig. 15a).^88^ The method was selective for Tyr in the presence of other unprotected alcohol-containing residues (Ser and Thr). However, other nucleophilic moieties (*e.g.*, N- and C terminal groups) need to be protected, thus restricting the range of applications for this reaction. Ociepa *et al.* reported an alternative alcohol selective phosphorylation method which utilizes a P(v) stable reagent (originally developed for the phosphorylation of oligonucleotides), named Ψ^o^ (69).^89,90^ The unique *O*-selectivity of 69 allows phosphorylation of Tyr containing peptides in DMF with the base 1,8-diazabicyclo[5.4.0]undec-7-ene (DBU) (70) upon hydrolysis of the Ψ^o^-loaded adduct in presence of amino groups, and other unprotected amino acids, such as His, Asn or Gln (Fig. 15b). However, Ψ^o^ shows a preferred selectivity towards aliphatic alcohols over the aromatic counterparts, with Ser being selectively phosphorylated in presence of Tyr (>15 : 1) and Thr (5 : 1). Thus, *O*-protection of Ser or Thr when targeting specific phosphorylation of Tyr is required. One limitation of phosphotyrosine-containing peptides is their poor *in vivo* stability due to the rapid cleavage of the phosphate group by phosphatases. Consequently, the synthesis of phosphotyrosine mimetics has emerged as a robust solution. Recently, Chen *et al.* developed a visible light photoredox/nickel-catalyzed cross-coupling method for the late-stage functionalization of peptides with phosphotyrosine mimetics, which offers a simpler alternative to previously reported methods utilizing Tyr-protected amino acids.^91^ Although this method does not selectively modify Tyr residues, it allows modification of peptides with or without side chain protections, making it highly versatile.

**Fig. 16:**
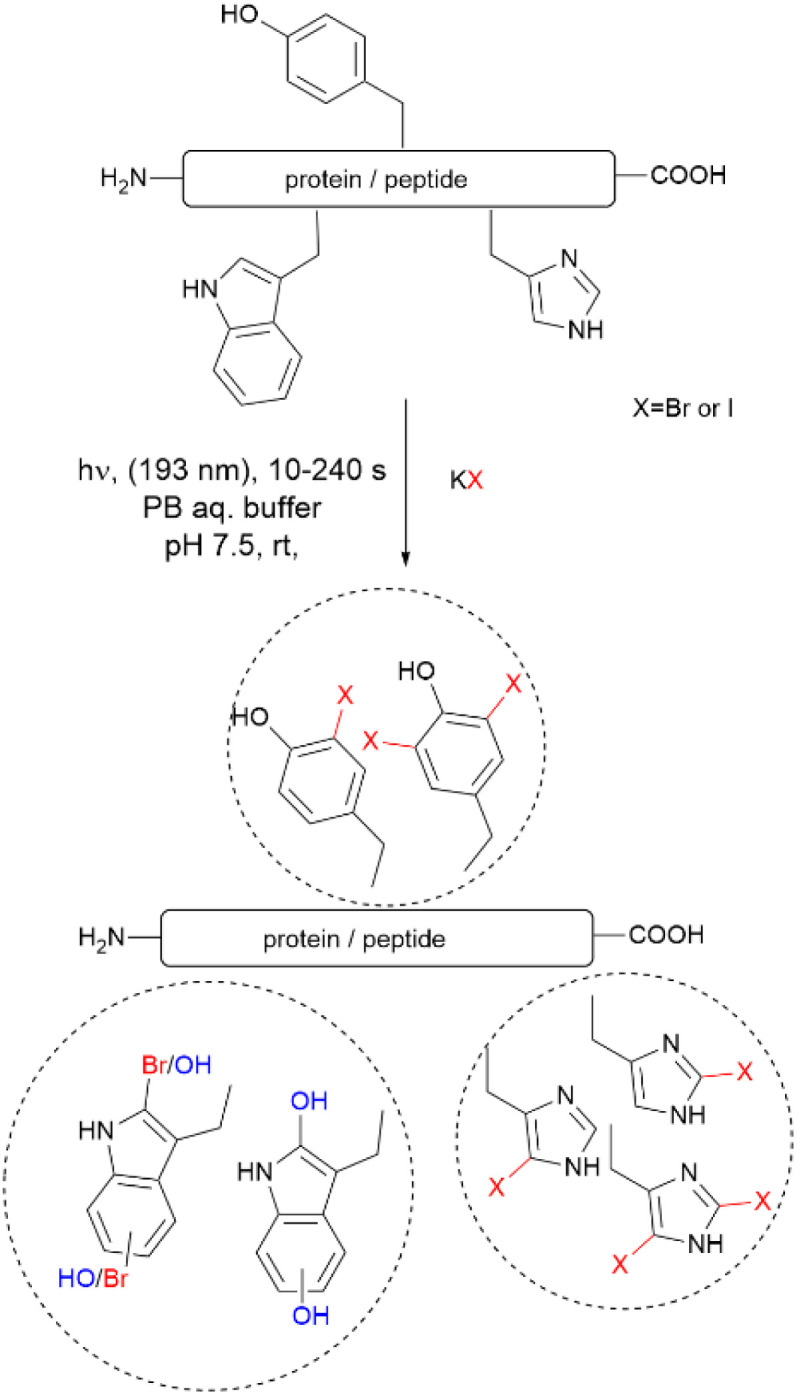
Photochemical bromination and iodination of Tyr residues in proteins and peptides.

Recently, Luo *et al.* reported a photochemical method to effect the bromination and iodination of peptides and proteins.^92^ The method avoids the usage of strong oxidants, metal catalysts, toxic halogenation reagents, or organic solvent typically required in traditional halogenation reactions. The reaction consisted of an ultra-short pulse irradiation at 193 nm of the protein or peptide in a phosphate buffer solution (pH 7.4) containing a halide salt. The result was the mono- and di-bromination of Tyr and His, bromination/oxidation of Trp or the mono- and di-iodination of Tyr and His (Fig. 16). The major drawback of this method is that it is not chemoselective to Tyr and yields a mixture of mono- and di-halogenated products. However, the method opens the opportunity to explore the effects of halogenation on the structure and bioactivity of proteins. The protocol is also of value for the chemospecific halogenation of synthetically prepared peptides and serves as a tool for structure diversification in the screening of bioactive relevant derivatives.

**Fig. 17:**
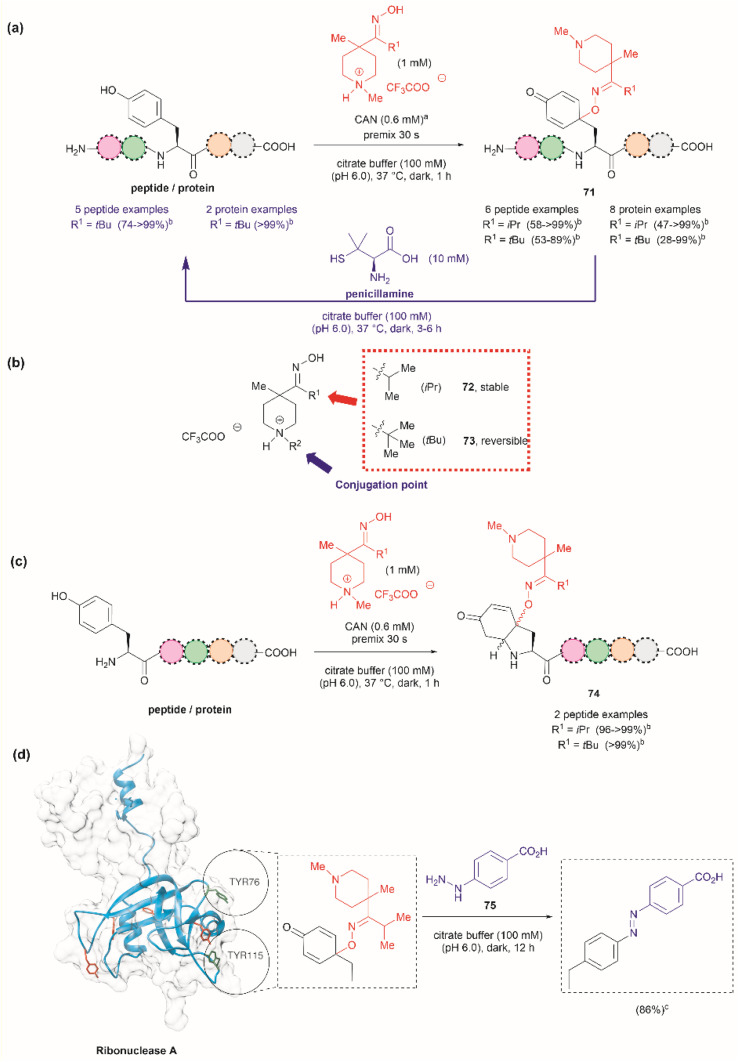
(a) Reaction conditions for the formation of cyclohexadienone adducts in Tyr-containing peptides/proteins and conditions for the reversible reaction to Tyr (blue arrow). ^*a*^Thiourea (5 mM) was added to minimize oxidation of proteins and peptides containing Met. ^*b*^Conversion determined by the HPLC peak area. (b) Structural modification points of the water soluble 4-methylpiperidinium oximes. (c) Post cyclization of the cyclohexadienone adduct in peptides containing Tyr at the N-terminus. (d) Introduction of benzodiazo substituents in ribonuclease A (PDB: 1A2W) cyclohexadienone adducts. For clarity the figure only shows one of the chains of the dimeric form of the protein. Tyr residues that are not modified by the iminoxyl radicals are colored in red and Tyr residues forming adducts with the radicals are labeled and shown in green. ^*c*^Calculated yield determined by LC-MS analysis.

Focusing on efforts to develop a synthetic protein modification protocol to reversibly control protein function, Maruyama *et al.* reported the chemoselective modification of Tyr residues with long-lived iminoxyl radicals.^93^ Initial screening with various oxime radical precursors showed that sterically hindered systems, such as di-*tert*-butyloxime and *tert*-butylisopropyloxime, resulted in higher yields than oximes with less bulky substituents in the reaction with a Tyr-containing peptide in presence of the strong one-electron oxidant ceric ammonium nitrate (CAN) in an acetonitrile/water mixture. This result was attributed to the lower bond dissociation energy of the O–H bond of the oximes with bulky substituents and their higher stability against deactivation reactions (*e.g.*, dimerization). In this reaction, the phenol of Tyr in peptides was oxidized to 1-iminooxycyclohexadienone 71, which aligned with similar reactions previously reported for other phenols containing electron-donating groups *para* to the hydroxy group (Fig. 17a).^94^

Implementing the same protocol in a strictly aqueous system led to a significant reduction in yield, largely due to the poor solubility of the oxime with bulky aliphatic substituents. To address this problem, Maruyama *et al.*^93^ cleverly substituted one of the *tert*-butyl units of the ketoxime with a methyl piperidinium group. Thus, a series of water-soluble oximes were screened from which two emerged as the most promising candidates for the iminoxyl mediated specific oxidation of Tyr-containing peptides: (1) isopropyl methyl piperidinium oxime (72), which gave stable cyclohexadiene adducts, and (2) *tert*-butyl methyl piperidinium oxime (73), which led to reversible systems (Fig. 17b). The design of the water-soluble oximes also allowed for the introduction of a conjugation site at the nitrogen atom of the methyl piperidinium unit (Fig. 17b). The optimized reaction conditions consisted of mixing the oxime with CAN in citrate buffer (pH 6.0) for 30 s to pre-form the iminoxyl radical and thus minimize protein degradation. The addition of the protein/peptide then followed, and the reaction was allowed to reach completion over 1 h at 37 °C in the absence of light. This protocol was successfully applied to several bioactive peptides and proteins with excellent conversions for most cases (Fig. 17a). The reaction was compatible with the side chains of most amino acids, except Met and Cys, which are oxidized. Moreover, Met oxidation was minimized by adding thiourea to the reaction mixture. Furthermore, it is important to note that when Tyr was the N-terminal residue, the formation of a diastereomeric mixture of cyclized product 74, as a result of an intramolecular conjugate addition of the N-terminal amino group to the cyclohexadienone, was observed (Fig. 17c).

The cyclohexadienone adducts formed with the *tert*-butyl methyl piperidinium iminoxyl radical slowly reversed to Tyr, a process that could be accelerated when the compound was exposed to light. This was attributed to the higher stability of the more substituted radical. This rearomatization reaction was proposed to occur *via* a thermally or photochemically promoted homolytic cleavage of the C–O bond with subsequent reduction of the resulting phenoxy radical. However, further mechanistic details are still pending. The rearomatization of the cyclohexadienone adducts was achieved in presence of penicillamine (Fig. 17a, blue reaction path) and was successfully applied to various peptide and protein substrates with good to excellent conversions (74–>99%). However, the reduction of cyclohexadienone to Tyr did not work in the case where the cyclized products were obtained when Tyr was located at the N-terminus or with oxytocin, in which the disulfide bond was cleaved, leading to multiple unidentified products.

The potential application of the cyclohexadienone to Tyr transformation was demonstrated in the reversible control of the function of the cyclohexadienone modified enzyme α-chymotrypsin and antibody trastuzumab. In both cases, a significant decrease in bioactivity was observed when Tyr residues were modified to cyclohexadienones, but this was regained upon reconversion to the Tyr residues with penicillamine.^95^ Unfortunately, the translation of the reversible chemical transformation described herein to living systems is unmet, but it could find future applications in the generation of cyclohexadienone based prodrugs that can be activated upon exposure to an anomalous reducing environment, such as the one found in some cancer tumors.^96^

Maruyama *et al.*^93^ also demonstrated the reaction of the cyclohexadienone adduct with phenyl hydrazines (75) for the generation of 4-azobenzene Phe peptides/proteins, as exemplified with ribonuclease A. Of a total of six Tyr residues in the enzyme, only two formed the cyclohexadienone adduct with the iminoxyl radicals (Fig. 17d).

**Fig. 18:**
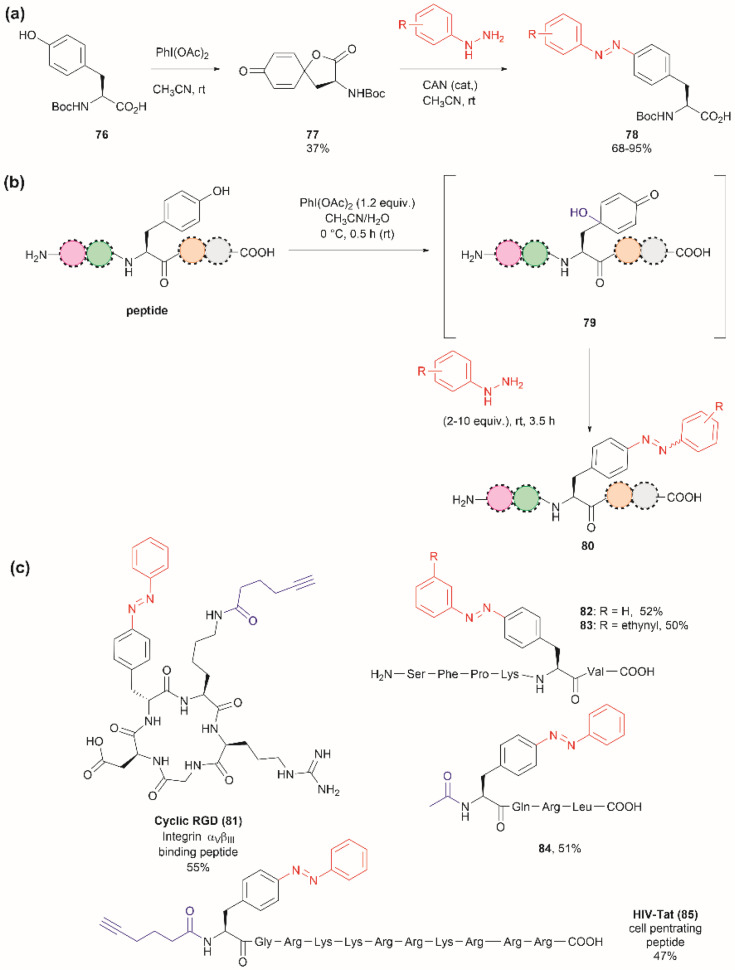
(a) Synthesis of azobenzene alanine reported by John *et al.*^97^ (b) Tyr specific functionalization of peptides with azobenzene moieties reported by Wang *et al.*^98^ (c) Examples of peptides functionalized with azobenzene using the method. The yields correspond to the trans isomer.

The introduction of azobenzenes into proteins and peptides is of interest given the light-responsive properties that these compounds provide based on their light-controlled *trans*–*cis* reversible isomerization. In 2015, John *et al.* reported the synthesis of azobenzene amino acids starting from Boc-Tyr-COOH (76) in a two-step protocol consisting of (1) the oxidation of the amino acid into a quinoidal spirolactone 77 with PhI(OAc)_2_, and (2) the formation of a 4-azo aryl substituted Phe 78 upon the reaction with phenylhydrazine in presence of catalytic amounts of CAN (Fig. 18a). The azobenzene amino acids were then incorporated into proteins in genetically modified bacteria.^97^ This reaction stands as a remarkable alternative to the reaction with typically unstable diazonium salts widely discussed in previous reviews.

Inspired by the work of John *et al.*,^97^ Wang and coworkers recently demonstrated that a similar synthetic protocol can be applied for the late-stage functionalization of Tyr-containing peptides.^98^ Wang *et al.* performed a one-pot reaction where they first added PhI(OAc)_2_ to the peptide dissolved in a 1 : 1 CH_3_CN/H_2_O mixture, followed by the addition of phenylhydrazine. Of note is the fact that Wang *et al.* demonstrated that the addition of CAN was not necessary. Thus, the reaction proceeded *via* a dearomatization-rearomatization path where Tyr is first oxidized to a 4-hydroxy-cyclohexadienone 79, which then reacts with phenylhydrazine to afford azobenzene-containing peptides 80 (Fig. 18b). The process was successfully applied to several simple protected dipeptides and a few more complex unprotected peptide systems (81–85, Fig. 18c). Wang *et al.* demonstrated that the process was chemoselective towards Tyr as no modification was observed in the side chains of peptides containing Arg, Asp, His, Lys, Met, Ser or Trp residues. However, the reaction yields of isolated products varied from low to moderate (20–63%), with the lowest yield being observed for a dipeptide containing Trp (BocNH-Trp-Tyr-CO_2_Me). While not explicitly indicated by the authors, it was evident that for peptides containing Tyr at the N-terminus the N-terminal amino group should be blocked as this can act as a nucleophile in an intramolecular Michael reaction with the cyclohexadienone. Furthermore, in our view, it would be difficult to compare the yields of the isolated product as reported by Wang *et al.* for the various solvent proportions tested during the optimization of the reaction conditions, as peptide substrate solubility differences appeared to play an important role. For instance, Wang *et al.* demonstrated that for the model testing system AcNH-Tyr-CO_2_Me, the reaction successfully proceeded in pure water, albeit in lower yield (*e.g.*, 44%) than the reaction performed in a 1 : 1 CH_3_CN/H_2_O mixture (*e.g.*, 66%). However, the differences in solubility of the reagents between each solvent system were not considered. Moreover, we would like to highlight that the use of pure water expands the potential application of the protocol to proteins, something not demonstrated by the authors of this work.

**Fig. 19:**
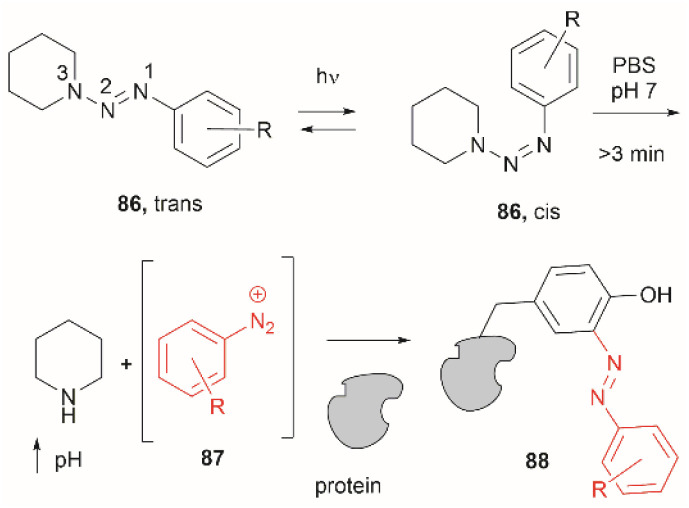
UV light driven isomerization of the triazene, promoting protonation at the N3 position and releasing diazonium for further protein modification at Tyr sites.

Two recent papers reported the selective functionalization of Tyr at the phenol C3 position with azobenzenes. The synthesis of phenyldiazenyl piperidine (86) was reported by Davis *et al.*^99^ These compounds, which are easy to synthesize *via* treatment of secondary amines with aryl diazonium ions, are bench stable and can release the aryldiazonium ion 87 upon activation with light *via* an isomerization mechanism that renders the triazene compounds more basic (Fig. 19). While the application of these compounds in producing an azobenzene-labelled protein 88 at a Tyr residue was demonstrated, it was also shown that under optimal conditions (pH 7–8) the liberated diazonium salt is photodegraded at longer irradiation times (>3 min 370 nm) hampering the reaction with aromatic residues.

**Fig. 20:**
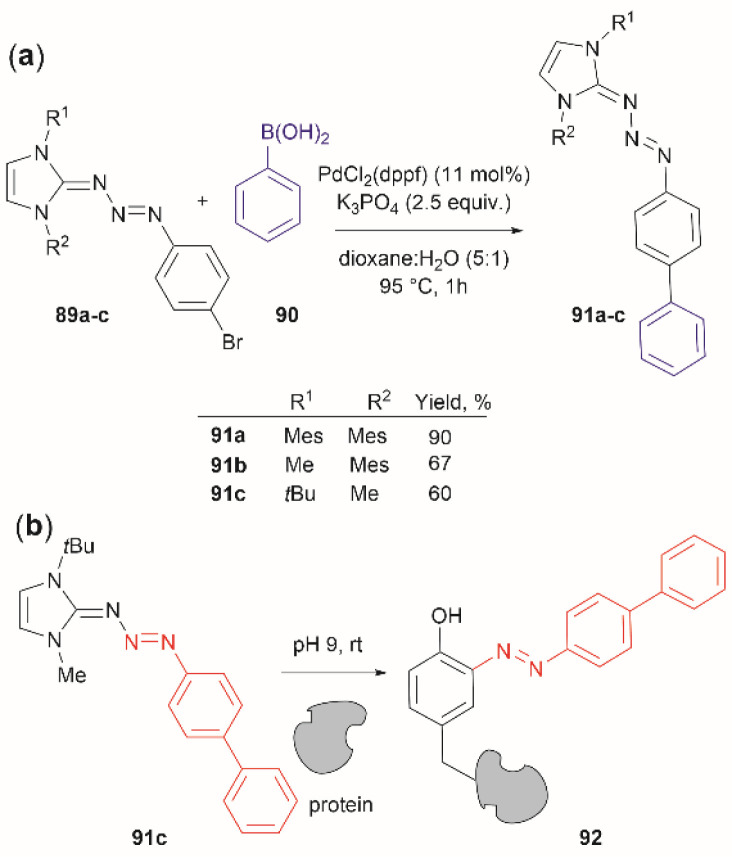
(a) Suzuki coupling of triazabutadienes *N*-protected derivatives. (b) Tyr-selective protein modification with functionalized triazabutadienes.

Triazabutadienes are stable scaffolds used to release diazonium ions under mildly acidic conditions.^100^ The synthesis and applications of triazabutadienes for specific derivatization and conjugation of Tyr residues have been widely discussed in the literature.^13^ Moreover, recently, the palladium-catalyzed Suzuki reaction of triazobutadienes has been reported.^101^ Thus, a series of diazoaryl triazabutadiene derivatives adorned with heterocyclic and aryl substituted rings were prepared. The main issue regarding the exposure of triazabutadienes to Suzuki coupling conditions was the stability of the triazabutadiene compound at high temperatures. The authors of this work reported that triazabutadienes 89a–c containing bulky *N*-substituents (*e.g.*, mesityl, *tert*-butyl) and a bromine in the aryl ring were stable to Suzuki high-temperature conditions and reacted efficiently with phenylboronic acid 90 to give diazoaryl triazabutadiene compounds 91a–c (Fig. 20a). The highest coupling yields were obtained using the *N*,*N′*-bismesityl triazabutadienes (91a), which presumably were the most stable reactants. However, the application of these aryl triazabutadienes in generating azobenzene-labeled protein 92 was demonstrated with a *tert*-butyl methylated triazabutadiene (91c) at pH 9.0 (Fig. 20b), which corresponded to the least stable compound reported in the work. The authors did not provide an explanation for their choice of labeling agent in the manuscript, but it is likely that the most stable triazabutadienes are also less susceptible to releasing the aryl diazonium reactant.

**Fig. 21:**
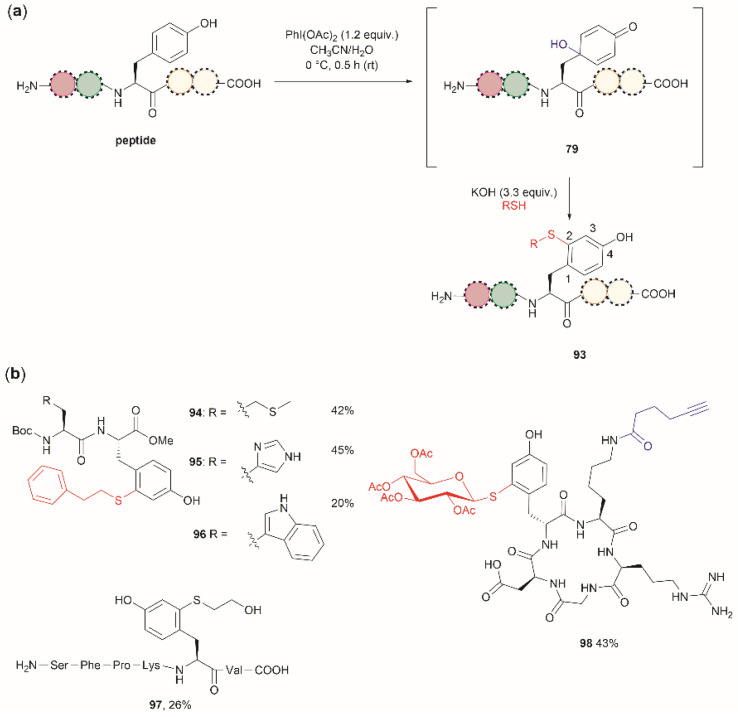
(a) Late stage 2-thiol functionalization of Tyr-containing peptides/proteins *via* a one-pot dearomatization-rearomatization reaction. (b) Examples of peptides functionalized at the 2-position of Tyr.

Wang *et al.* demonstrated that the dearomatization-rearomatization strategy can also be exploited for Tyr-specific peptide/protein functionalization with thiol-containing molecules through a thiol-Michael addition reaction (93, Fig. 21a).^102^ The most critical component for the optimization of this reaction was the base, which is required to neutralize the AcOH released from PhI(OAc)_2_. After screening different bases, the authors found that KOH was the optimal base at an amount of up to 3.3 equivalents. Higher amounts of base led to the destruction of the 4-hydroxycyclohexedienone 79. Worth mentioning is the finding that the reaction did not proceed in presence of organic bases (triethylamine or diisopropylethylamine). Furthermore, thiol functionalization of Tyr was shown to be chemoselective at the 2-position and the side chains of other residues (Arg, Asp, His, Lys, Met, Ser and Trp) remained unaltered when exposed to the reaction conditions. A drawback of this reaction, however, is the relatively low yields obtained for the isolated peptide products (<45%) and the lack of characterization of side products. A few examples of peptide functionalization were given for this process (94–98, Fig. 21b), as its most attractive application is in the field of peptide/protein bioconjugation.

One can further propose that the thiol–addition reaction presented by Wang *et al.*^102^ can also be applied to intramolecular cyclization reactions of native peptides, a process commonly termed “stapling”. Peptide stapling is an important tool for modifying the properties and structure of peptides and is widely employed in drug development. The method will be of value for those instances where Cys and Tyr are present in the same native sequence. Stapling *via* two different residues provides a means of enhanced reactivity control and positional selectivity over conventional stapling methods using the same type of residues (*e.g.*, Cys–Cys). However, a potential disadvantage of the proposed stapling protocol is the low frequency of Cys, which decreases the probability of finding Tyr and Cys residues at an appropriate distance within a native sequence.

**Fig. 22:**
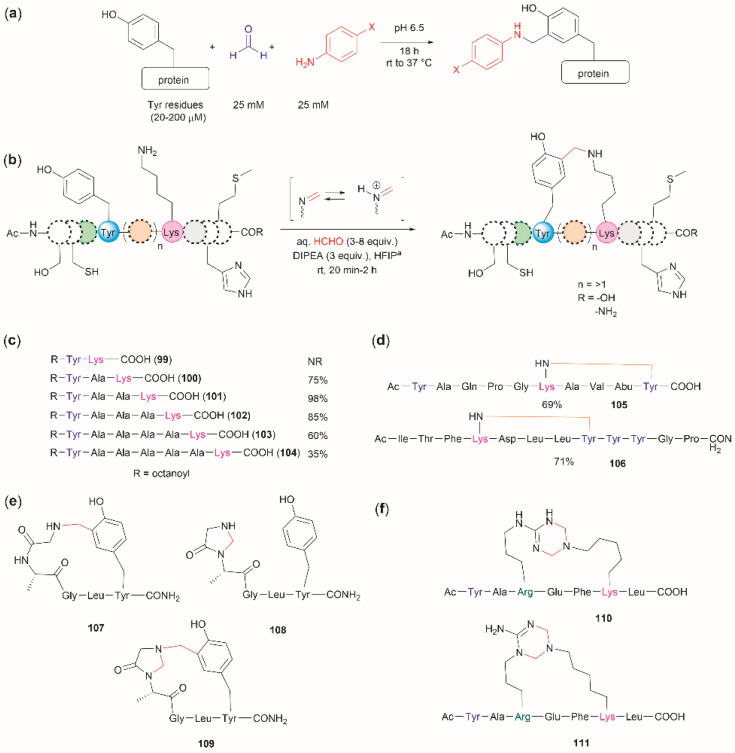
(a) Chemoselective Tyr protein functionalization with formaldehyde and aromatic amines as reported by Joshi *et al.*^105^ (b) Lys–Tyr stapling protocol described by Li *et al.*^106^ (c) Effect of the number of residues between Lys and Tyr in the efficacy of the stapling reaction after 1 h. (d) Peptide systems selected to exemplify the closest Lys–Tyr pair reactivity. (e) Products of the stapling between the N-terminal amino group and Tyr. (f) Formaldehyde mediated stapling reaction between Arg and Lys residues.

The cross-linking of Tyr (and also Arg, His and Trp) and Lys residues mediated by formaldehyde was recognized long ago.^103,104^ Lysine is a high-frequency amino acid commonly found on the surface of proteins, hence it is expected to appear more often in the neighbourhood of Tyr residues than Cys. Joshi *et al.* reported the selective functionalization of Tyr in proteins with aromatic imines generated *in situ* from the reaction between formaldehyde and arylamines (Mannich-type of reaction), both in large excess relative to the protein's Tyr residues (up to 100 times) (Fig. 22a).^105^ Yet, under these conditions Joshi *et al.* reported no interference in the presence of alkyl amines.

Inspired by the work of Joshi *et al.*,^105^ Li and collaborators recently described a formaldehyde-mediated crosslinking process involving Lys proximal to Tyr.^106^ The experimental highlights of the method are (1) the use of hexafluoroisopropanol (HFIP) as the solvent, and (2) the addition of only 3–10 equivalents of formaldehyde to the reaction mixture in presence of the organic base diisopropylethylamine (DIPEA). Under these conditions Li *et al.* demonstrated that peptides containing Lys and Tyr residues separated by at least one residue will crosslink by a proximity effect *via* a process involving formation of an imine between formaldehyde and the ε-amino group of lysine and a Mannich-type nucleophilic attack by the carbon-3 at the Tyr's phenol moiety on to the corresponding iminium species, which then rearomatizes after deprotonation by the base (Fig. 22b). Li and collaborators reported that the HFIP solvent played an important role in the completion of the reaction, which can be attributed to HFIP's strong hydrogen bond donor ability known to promote the activation of carbonyl substrates.^107^ Li *et al.* also demonstrated that the efficiency of the Lys–Tyr stapling reaction depended on the number of amino acids between Lys and Tyr. Thus, while the reaction did not proceed when Lys and Tyr were contiguous as in peptide 99 (Fig. 22c), the reactivity was optimal when the Lys and Tyr were separated by two to three residues and decreased as the number of residues between Lys and Tyr increased (100–104, Fig. 22c). Additionally, the authors also showed that for peptide sequences containing two or more Tyr or Lys with different separations (105, 106), cross-linking occurred at the closest pair (Fig. 22d). Considering these findings, the robustness of the reaction to the presence of other amino acids is more difficult to discern, since the authors failed to determine the efficiency of the Lys–Tyr stapling process in peptide systems where Tyr and the amino acid under assessment were separated by the same number of amino acids from Lys. Thus, the only conclusion that one could draw from the data reported by Li and collaborators is that the side chains of Arg, His and Met are not affected during the Lys–Tyr stapling when they are either contiguous to Lys or separated by more amino acids from Lys than the nearest Tyr reactive pair. The presence of a Cys distant to the [Lys–Tyr] fragment does not interfere with the stapling, but a Cys closer to Lys than Tyr to Lys competes for the formation of a linked product (–NH–CH_2_–S–). Fortunately, this product is easily hydrolyzed by running the reaction in presence of a small amount of water (5%), hence posing no further problem. In the case of Trp, it did not cross-link with Lys when simultaneously placed at the same amino acid distance from Lys as Tyr. However, a portion of the Lys–Tyr stapled product was modified with a methyl group at the *N*-heterocyclic atom of Trp. Further studies are needed to determine if Trp could cross-link with Lys when placed at a closer distance than Tyr or if the nature of the amino acids between the stapling pair could play some factor. There are two major issues concerning the Lys–Tyr stapling method. The first one is the formation of various cyclic side products 107–109 (Fig. 22e) formed with the N-terminal amino group when Tyr is near the N-terminus, which limits the application of the method to N-blocked peptides (*e.g.*, acetylated). The second issue is the selective formation of stapled product when Arg is near to Lys (see 110 and 111 in Fig. 22f).

**Fig. 23:**
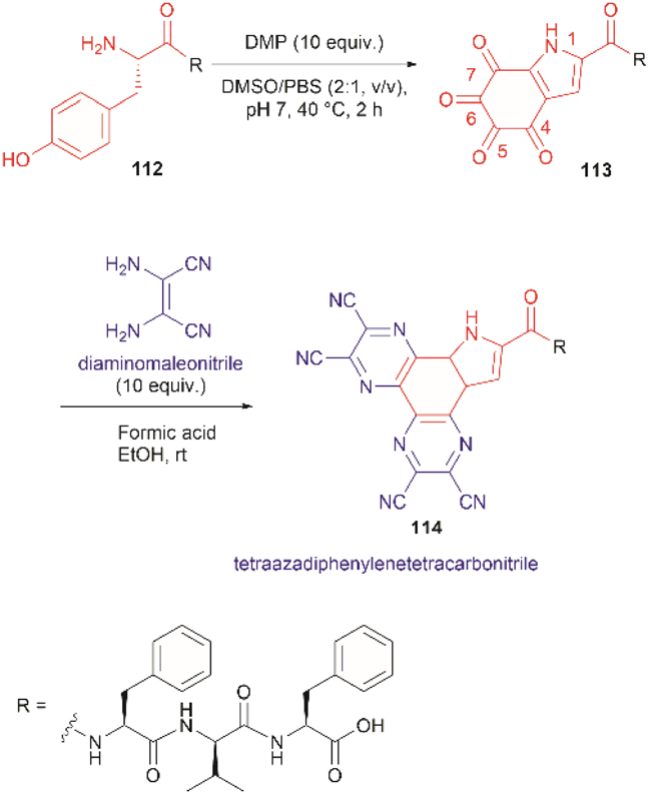
DMP-promoted Tyr-specific hyperoxidation of peptides and further derivatization with diaminomaleonitrile.

The use of hypervalent iodine compounds for Tyr selective peptide/protein functionalization/modification has surged in recent years, as exemplified by the oxidation of Tyr with PhI(OAc)_2_ discussed before. Another species that emerges as a potential alternative for the specific functionalization of Tyr residues in peptides is the TICA moiety (Fig. 2) resulting from the specific cleavage and hyperoxidation of Tyr with Dess–Martin periodinane (DMP) (Fig. 23).^108^ This tetraone is attractive for further peptide modification as it contains highly active ketones which can react with nucleophiles. An example of such reaction was provided by our research group in which peptide 113 containing the cyclohexatetraone unit was reacted with diaminomaleonitrile affording a tetraazadiphenylenetetracarbonitrile derivatized peptide 114, which can have a potential application in the field organic light emitting biomaterials.^109^ One can further envision the utility of the DMP-promoted Tyr-specific cleavage/hyperoxidation reaction as a tool to generate peptide fragments from proteins, and these fragments can then be assessed for bioactivity. The cyclohexatatraone unit of the bioactive candidates can further function as a catch and release handle (*ca.* through an imine–keto reversible process) or it can be derivatized to further expand the functionality space of the peptide fragments. Further research is needed to demonstrate the realization of these concepts.

**Fig. 24:**
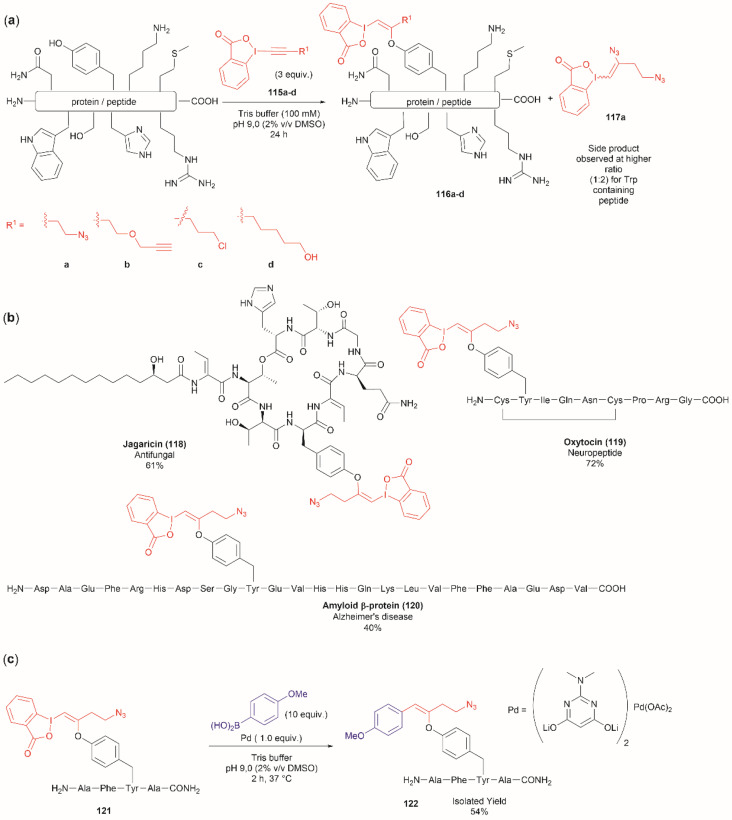
(a) Selective addition reaction of Tyr residues with ethynylbenziodoxolones. (b) Examples of bioactive peptides functionalized with a vinylbenziodoxolone. (c) Suzuki–Miyaura functionalization of vinylbenziodoxolone peptides.

Another recent report on peptide derivatization with hypervalent iodine compounds comprises the Tyr selective addition of the alkynyl bond of ethynylbenziodoxolones (115a–d) to give vinylbenziodoxolones (116a–d).^110^ Reaction conversion was highest in Tris buffer (pH 9.0) in accord with the higher nucleophilicity of the phenoxy group of Tyr (Fig. 24a). This method is robust as it can be applied to unprotected peptides/proteins in aqueous media and is independent of the position of Tyr within the sequence. Except for the thiol group in Cys, the side chains of most nucleophilic amino acids (Arg, Asp, His, Met, Ser, Thr, and Trp) did not interfere in the reaction. However, it is important to mention that when Trp was present in the peptide sequence an increase in the formation of a decomposition product (117a) derived from the ethynylbenziodoxolone reactant 115a was observed and also a decrease in yield was noted for an Asn containing peptide. The method was applied successfully to several Tyr-containing bioactive peptides giving the vinylbenziodoxolone-peptide products 118–120 in moderate to good conversions (40% to >90%) (Fig. 24b). The protocol was also tested in proteins (albeit requiring further individual optimization of the reaction conditions) wherein the derivatization of solvent accessible Tyr was detected.

A relevant feature of the vinylbenziodoxolone peptides described above is that they can be functionalized further, or they can be used as a linker for bioconjugation. An example of functionalization is the Suzuki–Miyaura cross-coupling of vinylbenziodoxolones peptide 121 with boronic acids shown in Fig. 24c. When an azide group is also present in the vinylbenziodoxolone unit of 121, bioconjugation *via* strain-promoted alkyne–azide cycloaddition can be performed.

#### 3.1.2. Genetically encoded Tyr-modified residues

Genetic code expansion is a powerful strategy for site-specific protein modifications, complementary to chemical methods. Hence, recent developments in the area for the specific modification of Tyr will be discussed next.

**Fig. 25:**
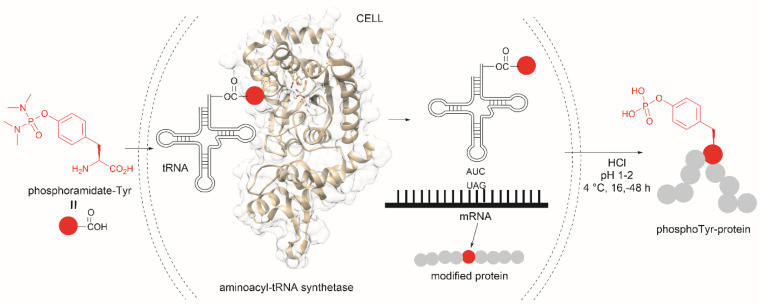
Genetic encoding of phosphoramide-Tyr into proteins and subsequent deprotection results in the site-specific formation of native phosphotyrosine-modified proteins.

The fundamental genetic modification required for the specific incorporation of non-canonical Tyr-modified amino acids into proteins is to introduce a new tRNA/aminoacyl-tRNA synthetase (aaRS) pair which is orthogonal to endogenous tRNA-aaRS into cells.^111,112^ This orthogonal aaRS specifically binds the non-canonical Tyr- amino acid and then catalyzes its esterification with tRNA. The tRNA must deliver the non-canonical amino acid in response to a unique codon that does not translate into any canonical amino acid. This is typically a non-sense amber termination codon (*e.g.*, UAG), with limited use in the host cell. The production of proteins incorporating the non-canonical Tyr residue is carried out by growing the genetically modified cells in media enriched with the non-canonical amino acid (usually chemically made) at relatively high concentrations to reach intracellular levels appropriate for incorporation into proteins. However, deficient intracellular uptake of the non-canonical amino acid and/or poor protein incorporation are the major drawbacks of this technology, which result in low yields of the modified protein. Low intracellular uptake of non-canonical amino acids is prominent in the case of highly hydrophobic, highly charged, or polar molecules, such as phosphotyrosine, which is also susceptible to cleavage by cellular phosphatases. A solution to the particular case of phosphotyrosine is the introduction of a charge-neutral and stable phosphoramidate-Tyr analogue, which can be hydrolysed to the phosphate after its incorporation into modified proteins (Fig. 25).^113^ However, the complex post-purification protocols limit the efficiency of the methodology.

**Fig. 26:**
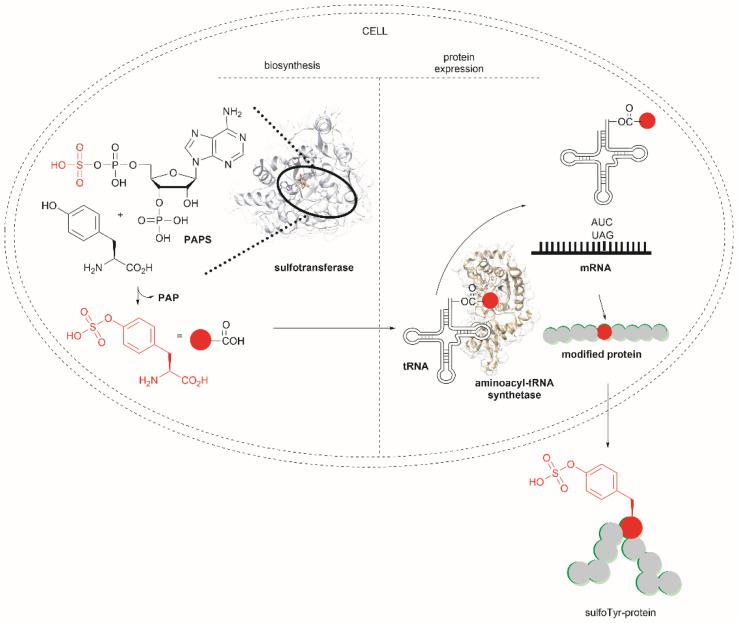
Schematic representation of the biosynthesis of Tyr-*O*-sulphate and its site-specific incorporation into a green fluorescent protein in cells.

Another strategy used to overcome the limited intracellular uptake of non-canonical amino acids relies on the engineering of autonomous cells, which are cells that can biosynthesize and then incorporate the desired non-canonical unit. A recent successful example of the application of this technique is the biosynthesis of tyrosine-*O*-sulfate by prokaryotic and eukaryotic modified cells.^114^ In this case the cells were encoded with a cytosolic sulfotransferase, which uses an active form of sulfate, 3′-phosphoadenosine-5′-phosphosulfate (PAPS). The enzyme is specific for Tyr where the sulfation reaction comprises an SN2- type nucleophilic attack of the phenoxide of Tyr to PAPS (Fig. 26). Under optimal conditions it was demonstrated that the biosynthetically produced sulfated Tyr can be genetically incorporated into proteins in *Escherichia coli* (*E. coli*) in response to the amber codon. Thus, a Tyr-sulfated green fluorescent protein was produced by an autonomous *E. coli* strain in a 5.67 mg L^−1^ yield. This yield was nearly 4 times larger when compared with the 1.5 mg L^−1^ yield of the same protein produced by feeding the bacteria with 1 mM exogenous tyrosine-*O*-sulfate. Other successful examples of this technology include the site-specific incorporation of *O*-methyltyrosine,^115^ and 3,4-dihydroxyphenylalanine (l-DOPA).^116,117^

l-DOPA is another non-canonical amino acid, which results from the posttranslational modification of Tyr. l-DOPA is found in biopolymers in nature (*e.g.*, mussel foot proteins, and tubeworm adhesive), whose properties have attracted attention for the development of new biomaterials.^118^ Additionally, l-DOPA is a target residue for protein/peptide functionalization and bioconjugation. There are several reports on the biosynthesis of l-DOPA and its incorporation into proteins by engineered autonomous cells. Kim *et al.* reported the biosynthesis of l-DOPA from catechol, pyruvate, and ammonia in *E. coli* together with its incorporation into proteins.^116^ However, the need for exogenous addition of the precursor chemicals of l-DOPA and suboptimal selectivity for l-DOPA of the orthogonal aaRS limit the application of this method. The latter issue is attributed to the small structural steric differences between Tyr and l-DOPA, complicating their discrimination by the orthogonal translation machinery, and resulting in the misincorporation of Tyr. Thyer and collaborators introduced a biosynthetic path to prepare l-DOPA from Tyr with an oxygenase-FAD reductase pair and developed a new orthogonal translation machinery with higher affinity for l-DOPA over Tyr for the site-specific incorporation of l-DOPA in proteins.^117^ However, while co-expression of the oxygenase and an improved aaRS in an *E. coli* strain allowed the site-specific incorporation of l-DOPA in proteins, production yields were not reported. More recently, Chen *et al.* reported the efficient biosynthesis of l-DOPA from Tyr using a hydroxylase together with a tetrahydromonapterin (MH4) cofactor recycling enzyme. The authors also further screened previously reported aaRS/tRNA pairs and selected one that led to the production of pure DOPA-containing proteins without the misincorporation of tyrosine (a chimeric pair of pyrrolysyl-tRNA synthetase/tRNA pair and PheRS/tRNA from human mitochondria).^119^ With these vectors they generated an autonomous *E. coli* strain that produced a superfolder green fluorescent protein-l-DOPA in a 3.1 mg L^−1^ yield, while control cells fed with 9 mM DOPA gave a yield of 2.5 mg L^−1^.^120^ The study demonstrated for the first time an improvement in the incorporation of l-DOPA when using the biosynthetic path.

### 3.2 Tyr bioconjugation

Bioconjugation is the formation of a covalent bond between a biomolecule (*e.g.*, proteins/peptides, DNA, *etc.*) and another molecule that typically presents a specific desired property (*e.g.*, fluorophores, drugs, *etc.*). Important applications of bioconjugation are in the burgeoning areas of biological probe construction,^121^ targeted delivery agents^122^ and biomaterials.^123^

#### 3.2.1. Enzymatic oxidative conjugation

**Fig. 27:**
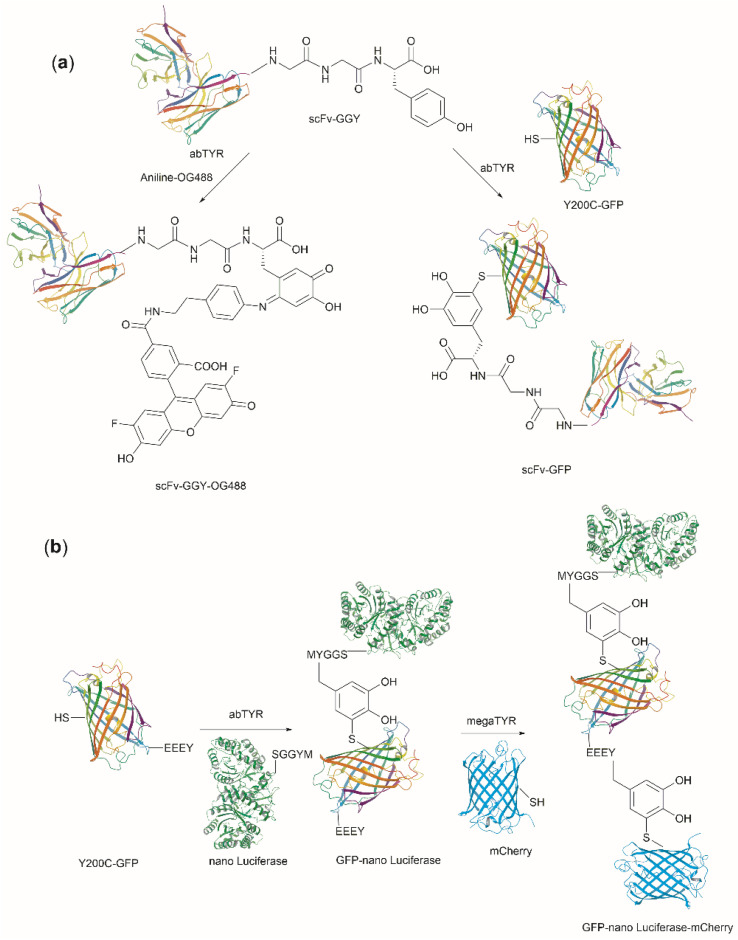
(a) Tyrosinase-enabled bioconjugation of scFV (PDB ID: 1FVC) with aniline-containing dyes and Y200C-GFP (PDB ID: 5b61). (b) Protein trimer GFP-nano Luciferase (PDB ID: 7SNR)-mCherry (PDB ID: 4ZIN) assembly using CDSAT.

Conjugation of proteins/peptides induced by enzymatic oxidation is a green methodology of great interest in the development of bioconjugated systems. Francis *et al.* have reported a tyrosinase-mediated oxidative coupling strategy for Tyr-selective bioconjugation at protein C- or N-termini.^124^ A commercially available mushroom tyrosinase from *Agaricus bisporus* (abTYR) was found as a highly selective catalyst to facilitate oxidation of Tyr to an *ortho*-quinone under mild conditions (phosphate buffer, pH 6.5, 4 to 23 °C) without collateral oxidation of other sensitive residues (Cys and Met). The generated *ortho*-quinone species can couple with various nucleophiles *via* Michael addition. Aniline or cyclic-amines were discovered as the best coupling partners because they can react with the *ortho*-quinone in a favorable manner over other intramolecular nucleophiles present in the protein, thus leading to a clean and robust conjugation process. Apart from the high chemoselectivity, this technology has also exhibited prominent regioselectivity by targeted Tyr conjugation at protein termini while leaving other non-solvent exposed Tyr residues untouched. This unique attribute has proven important in retaining protein biological function and generation of a homogeneous conjugate system. Using this technology, one fluorescent-tagged aniline cargo molecule was quantitively conjugated to the C-terminal Tyr tag of protein L and the single chain variable fragment (scFv) of Trastuzumab antibody, which comprises multiple Tyr residues in its structure (left path, Fig. 27a). It is noteworthy that all the protein substrates used in this study did not contain any Cys residues on the protein surface as the thiol group of Cys also participates in the reaction with quinone species.

To further investigate the reaction between Cys and *ortho*-quinone, a separate study was carried out by the Francis group. From this study, it was revealed that the thiol group of Cys can selectively couple with the *ortho*-quinone generated from tyrosinase-mediated oxidation of Tyr without affecting other residues in the protein.^125^ An unusual 1,5-disubstituted catechol product was formed after the thiol addition to *ortho*-quinone, which is distinct from the 1,6-disubstituted *ortho*-quinone moiety generated from the reaction between quinone and amine nucleophiles. Encouraged by the high selectivity of this Cys–Tyr cross-linking reaction, a series of bioactive peptides containing an N-terminal Tyr were conjugated to the Y200C green fluorescent protein (Y200C-GFP) and N87C MS2 viral capsid using this tyrosinase oxidation/thiol addition protocol. The resulting conjugates were stable in a variety of buffers for long-term storage and showed superior plasma stability compared to the widely used thiol-maleimide counterpart. Furthermore, this novel conjugation technology was also exploited to attach two copies of a cell-penetrating peptide to an RNA-guided DNA endonuclease CRISPR-Cas9 bearing two solvent-exposed Cys on the surface. This construct improved cellular uptake of the protein and can be used for targeted delivery of therapeutics. Finally, the capability of this chemistry for protein–protein conjugation was illustrated. A HER2-binding scFv with a tyrosine tag at its C-terminus was efficiently combined with Y200C-GFP *via* a Cys–Tyr crosslink (right path, Fig. 27a). The fluorescent-labeled scFv was then applied to capture HER2-expressing cells from a mixture of HER2+ cells and HER2-negative cells.

In a follow-up study on protein–protein conjugation activated by tyrosinases, the use of abTYR was limited by the difficulty of recombinant production and the restricted substrate scope. Therefore, Mogilevsky *et al.* reported the discovery of a new tyrosinase from *Bacillus megaterium* (megaTYR), that presented a broad substrate tolerance compared with abTYR.^126^ Further protein engineering of megaTYR led to the production of a panel of megaTYR mutants with different substrate charge preference. By exploiting this charge preference, biorthogonal activation of different Tyr residues within proteins could be achieved based on their charge context. This enabled the successful construction of a protein trimer through a charge-directed sequential activation of tyrosine residues (CDSAT) (Fig. 27b).

Given the increasing use of tyrosinase for protein conjugation, separation of protein-enzyme mixtures after conjugation poses another obstacle to its practical application, especially when reaction scale-up is needed. A heterogeneous system comprising tyrosinase immobilized on magnetic beads was lately reported by Ji *et al.* to ease the purification of protein conjugates post oxidation with the enzyme.^127^ The enzyme bound to a solid phase could be readily removed from the reaction mixture by a magnetic device and was recyclable after a washing cycle with buffers. The authors then demonstrated the applicability of this reaction system in a robust peptide and protein conjugation process with aniline derivatives.

**Fig. 28:**
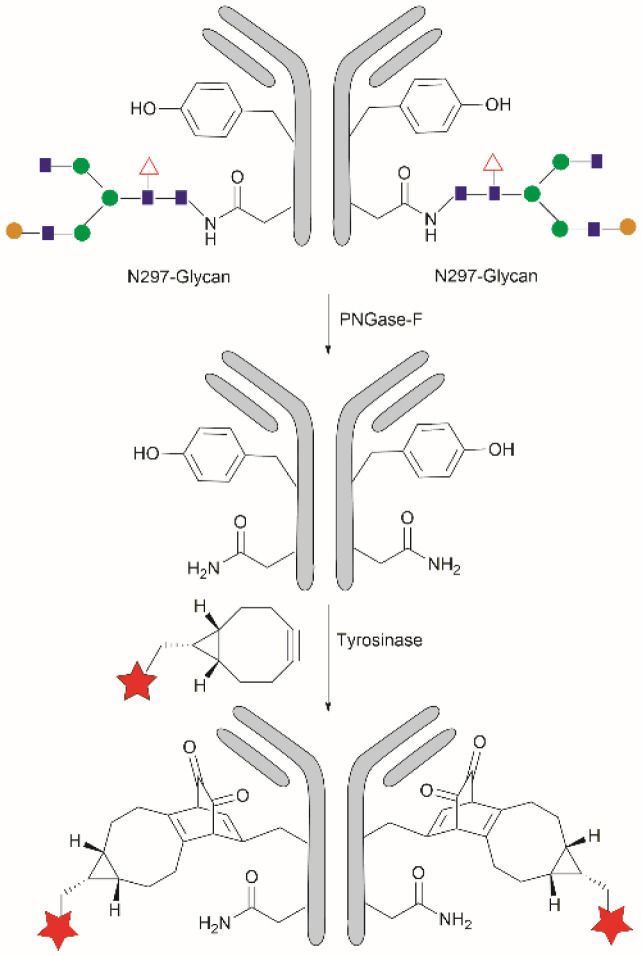
Homogeneous conjugation of antibody using chemoenzymatic tyrosine click chemistry.

Considering that one of the main issues for current bioconjugation methods is the production of heterogeneous systems (*i.e.*, where more than one site is covalently modified), the development of methods that can yield homogeneous systems is highly pursued, yet rarely accomplished. Recently, Bruins *et al.* reported a two-stage protocol for the selective conjugation of toxic drugs to antibodies, which can generate homogeneous antibody-drug conjugates.^128^ This method comprised antibody enzymatic Asn-deglycosylation to selectively expose Tyr residues in its proximity, followed by tyrosinase-mediated oxidation of Tyr and subsequent conjugation of a toxic payload *via* strain-promoted click chemistry with the *ortho*-quinone (Fig. 28). Using this technology, the generation of antibody–drug conjugates (ADCs) with precisely controlled drug-to-antibody ratio (DAR) 2 or 4 was effected by labeling of the deglycosylated trastuzumab with a series of small molecule payloads with >90% efficiency.

#### 3.2.2. Chemical conjugation

**Fig. 29:**
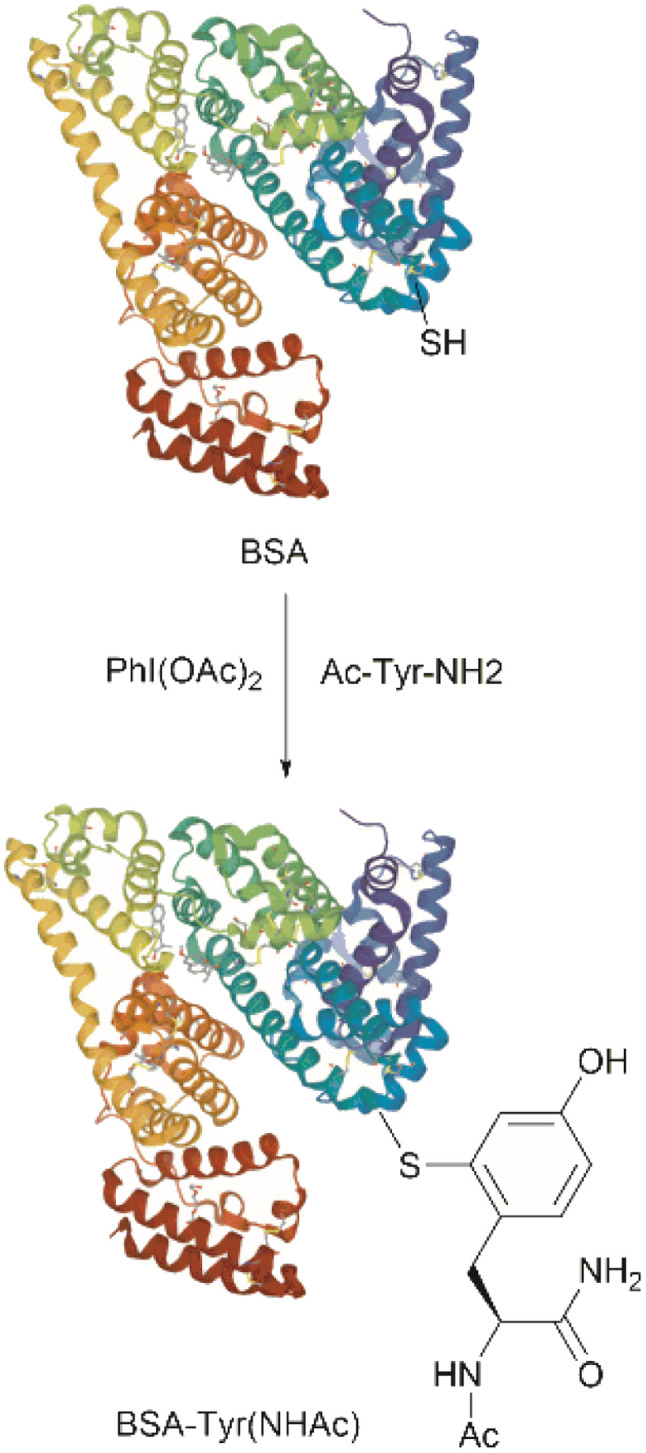
Conjugation of BSA (PDB ID: 4F5S) with Ac-Tyr-NH_2_ using PhI(OAc)_2_.

Residue-specific modification of peptide/proteins by chemical reagents offers another invaluable tool for bioconjugation. The abovementioned Tyr-selective functionalization *via* dearomatization–rearomatization strategy has been demonstrated applicable to peptide and protein conjugation.^102^ Compounds with a free thiol group could be chemoselectively coupled to the peptides at Tyr sites *via* thiol Michael addition with the generated 4-hydroxy-cyclohexadienone moiety. On the other hand, pre-activation of Tyr-containing peptides to form the 4-hydroxy-cyclohexadienone species could also serve as an electrophilic reagent for Cys-selective protein conjugation. This concept has been illustrated by the selective conjugation of Ac-Tyr-NH_2_ to bovine serum albumin (BSA) which comprises one surface-exposed cysteine (Fig. 29).

**Fig. 30:**
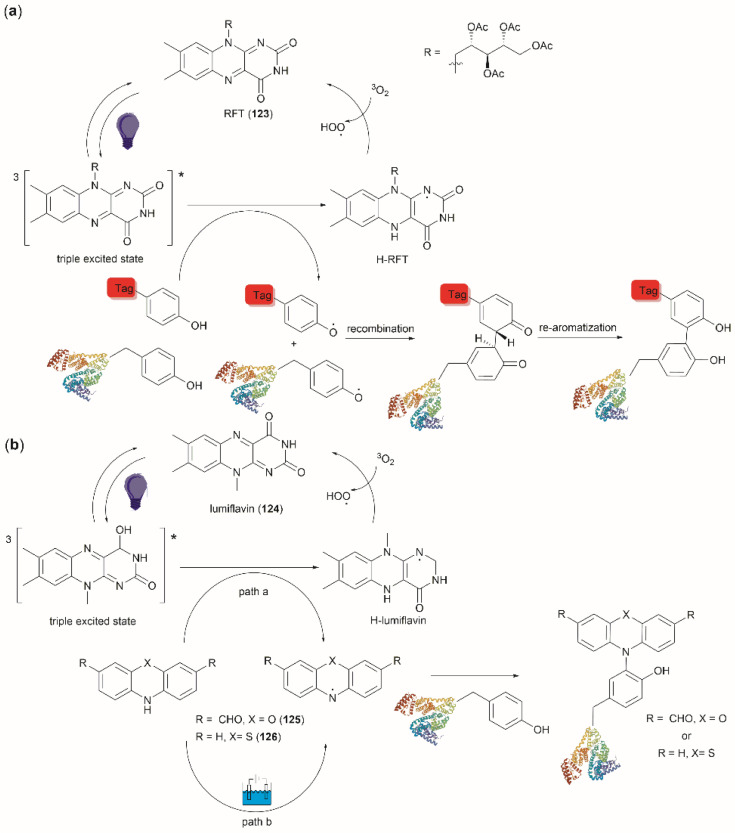
Photochemical protein conjugation at Tyr sites with (a) phenol-containing tags and (b) phenoxazine/phenoxazine tags.

The past several decades have witnessed the increasing use of photoredox catalysts for the development of site-selective, functional-group-specific conjugation of proteins due to their biocompatibility and facile use under mild conditions. Fadeyi's group recently reported the use of a flavin-based cofactor, riboflavin tetraacetate (RFT, 123), as a photocatalyst to initiate the Tyr-specific protein conjugation with a phenol-containing tag.^129^ A C–C bond is formed selectively between the phenol rings of Tyr and the tag molecule at the orthogonal position of the phenol. A study of the reaction mechanism showed that this conjugation process started with photoexcitation of the flavin catalyst to its singlet excited state by blue LED illumination, which was followed by the intersystem crossing to produce the triplet excited state. The triplet excited state of flavin then oxidized both the Tyr in the protein and the phenol group in the tag, generating two molecules of phenoxyl radicals. These two radicals could further undergo radical recombination and subsequent rearomatization to yield a C–C cross-linking between the two phenol rings (Fig. 30a). The authors have showed that this technology found applications in cell–cell interaction profiling using a single-domain antibody-based RFT conjugates targeting the proteins of interest on the cell surface (*vide infra*).

Another water-soluble photocatalyst, lumiflavin (124), was employed by MacMillan's group to catalyze the Tyr-selective peptide/protein conjugation with the phenoxazine dialdehyde (125) tag (Fig. 30b).^130^ Triggered by blue-light irradiation (path a), a phenoxazinyl radical was generated after oxidation of the phenoxazine tag by the triplet-excited lumiflavin. This electrophilic N-centered phenoxazinyl radical could react with the electron-rich phenol group of Tyr in a selective manner, yielding a C–N bond between the protein and the tag (path a, Fig. 30b). After optimization of the reaction conditions using a small peptide, this Tyr-specific conjugation method was applied to a collection of twelve proteins with the size ranging from 5.8 to 77.0 kDa. The results suggested that all tested protein substrates were successfully labelled with the phenoxazine tag with good to excellent conversion rates (49–100%). Furthermore, it was highlighted that this conjugation approach significantly outperformed other traditional methods in terms of the extraordinary single-site-specificity for proteins bearing multiple Tyr residues. The author demonstrated a remarkable site-selective process with the labeling of one Tyr residue of serotransferrin from a total of 26 Tyr residues available in this protein. It was proposed that this site-selectivity was attributed to the unique micro-environment where the Tyr residue is located. Tyr residues that are buried deep and associated with potential cation–π interactions are disfavored for conjugation as a result of steric and electronic factors. In addition, this phenoxazine tag conjugated to proteins could be further bioorthogonally derivatized with other useful functional moieties, including a fluorescent dye, a biotin or an azide/alkyne, *via* two aldehyde handles. This functionalization expands the applicability of this bioconjugation method for use in different scenarios. Similar to the phenoxazine tag, a phenothiazine tag 126 has also been reported to selectively label peptides and proteins at Tyr sites.^131^ Instead of being driven by light irradiation, the activation of phenothiazinyl radical was achieved by electrochemical oxidation (path b, Fig. 30b). High chemo- and site-selectivity selectivity, as well as high conversion rate, were also observed for peptide/protein labeling using phenothiazine.

**Fig. 31:**
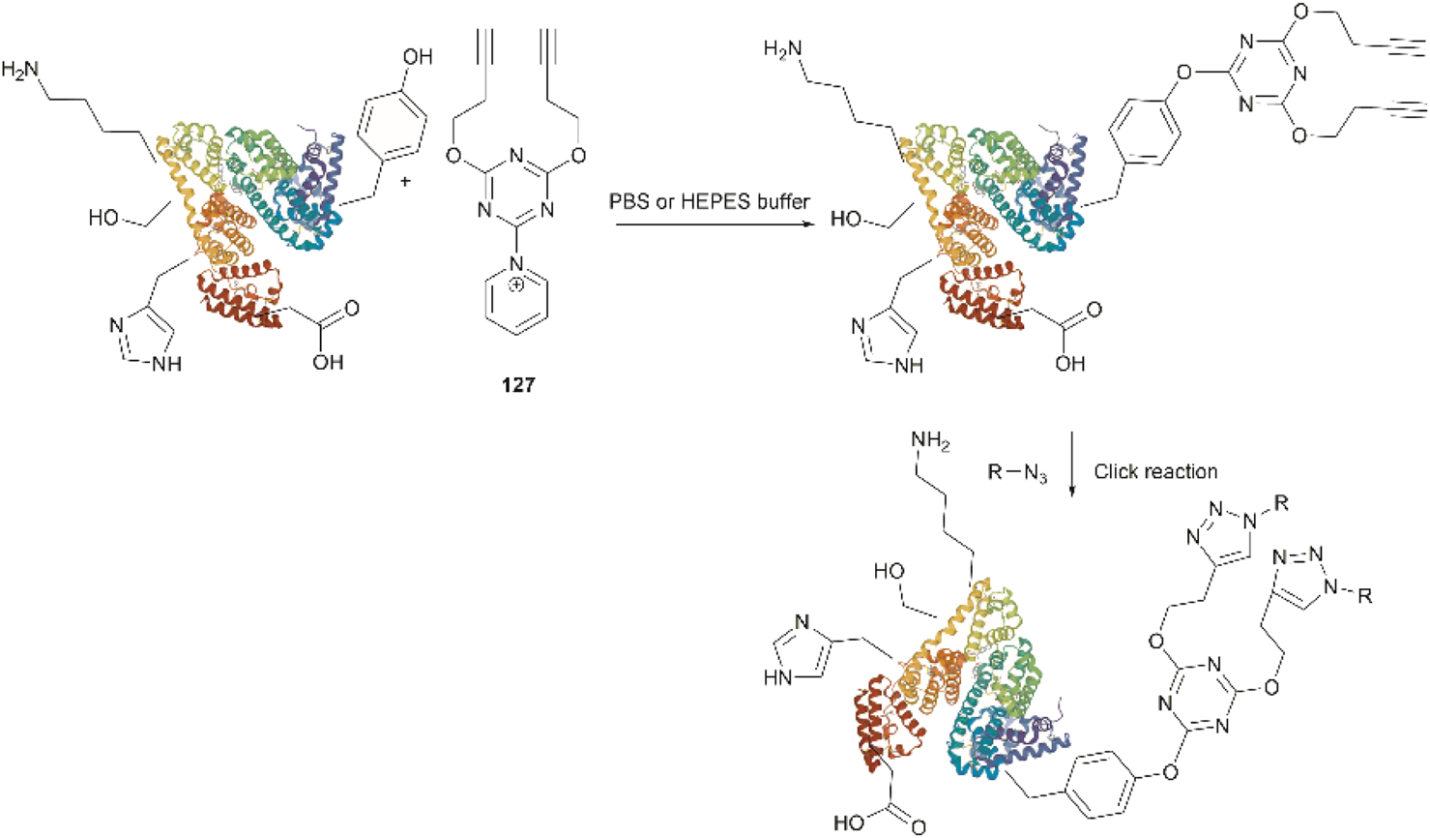
Tyr-selective protein labeling using TPC reagent.

Jiang *et al.* recently disclosed the identification of a novel 1,3,5-triazine-pyridine (TPC) reagent (127) for Tyr-specific protein conjugation (Fig. 31). The phenol side chain of Tyr was selectively modified by 127 at 30 °C under neutral conditions (pH 6.8), generating an *O*-1,3,5-triazinyl Tyr residue in the protein (Fig. 31). High residue selectivity was observed for this reaction but pre-blocking of Cys is required. It was demonstrated that a TPC reagent functionalized with two alkyne handles can be employed to effect chemoselective tyrosine labelling in the whole proteome of HeLa cells. Furthermore, TPC reagents were demonstrated to have no adverse effects on cell viability and proliferation, thereby enabling their use in live cell labeling and comprehensive tyrosine mapping within living cells.^132^

## 4 Applications

In order to offer a comprehensive perspective on the practical applications of Tyr-specific cleavage, modification/functionalization, and bioconjugation, this section will delve into recently published examples in the field.

**Fig. 32:**
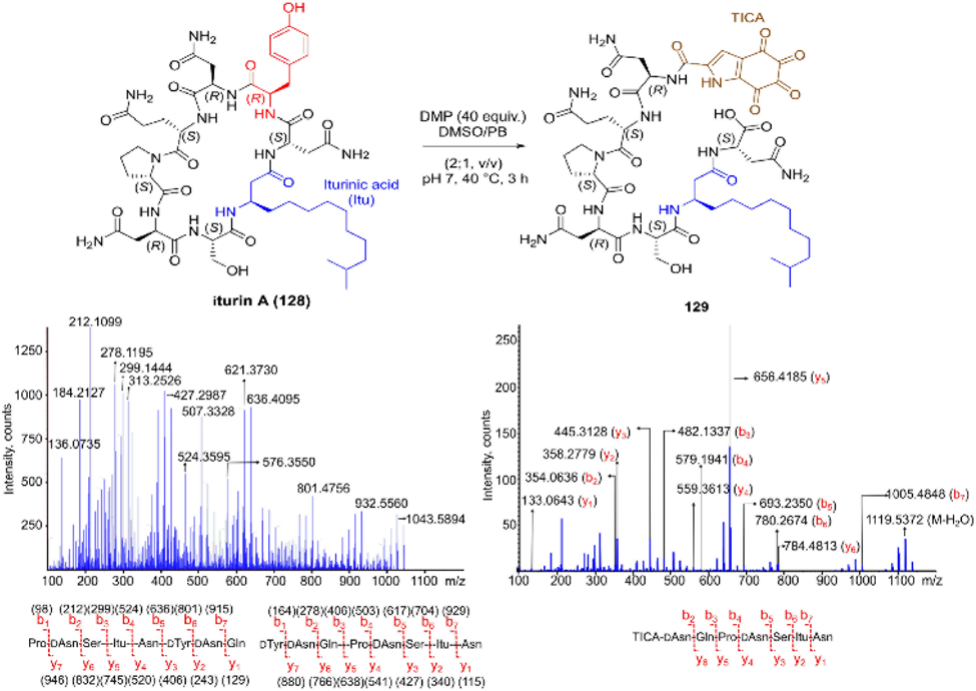
(Left column from top to bottom) Cyclic structure of inulin A, MS/MS spectrum of inulin A and theoretical b-type and y-type fragment ions obtained from ring-opening reactions at Gln–Pro and Asn–Tyr. (Right column from top to bottom) Linearized peptide structure of inulin A after Tyr-selective cleavage and hyperoxidation with DMP, MS/MS spectra of linearized peptide and experimentally observed b-type and y-type fragment ions. The shown high-resolution mass spectra were obtained using similar conditions on a QSTAR XL quadrupole-time-of-flight mass spectrometer.

Cyclic peptides derived from natural products are becoming an important source of novel pharmaceuticals.^133^ However, sequencing, and structural identification of novel cyclopeptides requires relatively large amounts of materials (∼mg) for analysis by nuclear magnetic resonance. Tandem mass spectrometry (MS/MS) is an attractive alternative for the sequence elucidation of newly discovered cyclic peptides when smaller amounts of material are available. Moreover, typically MS/MS spectra of cyclic peptides present complex fragmentation patterns derived from multiple ring-opening reaction sites, making their structural interpretation cumbersome. Further complications arise from non-ribosomal cyclopeptides for which protein databases are inapplicable. One relevant application of DMP mediated selective cleavage at Tyr residues is the linearization of cyclic peptides, which in turn simplifies the interpretation of the corresponding MS/MS spectra. Fig. 32 shows an example of this application for the case of iturin A (128), which is a potent antifungal non-ribosomal peptide containing a β-lipoamino acid residue, produced by various species of *Bacillus* bacteria.^134^ On the left side of Fig. 32, the cyclic structure of inulin A with the corresponding complex MS/MS spectrum are shown (top left and bottom left). Theoretical b-type and y-type fragment ions dissociated from ring-opening reactions at Gln–Pro and Asn–Tyr (left bottom panel) are also indicated to exemplify the origin of multiple fragmentation sites.^135^ On the right side of Fig. 32 one can see the structure of linearized iturin A (129) after Tyr selective cleavage with DMP (top right) bearing the characteristic TICA moiety. In this case, one can see a simple MS/MS spectrum, which signals can be easily assigned to the corresponding structural fragments of the linearized peptide.

Within the field of protein/peptide structure elucidation, Cui *et al.* reported the design, synthesis and application of a Tyr residue-specific, electrochemically cleavable cross-linker for probing the 3D structure of proteins using mass spectrometry.^136^ The general process consists of covalently attaching a cross linking unit to specific residues in a protein. The cross-linked protein is then enzymatically digested, and the resulting cross-linked peptides analyzed by liquid chromatography-tandem mass spectrometry (LC-MS/MS). A control experiment is also carried out in which the unmodified protein is digested, and the digest analyzed by LC-MS/MS.

**Fig. 33:**
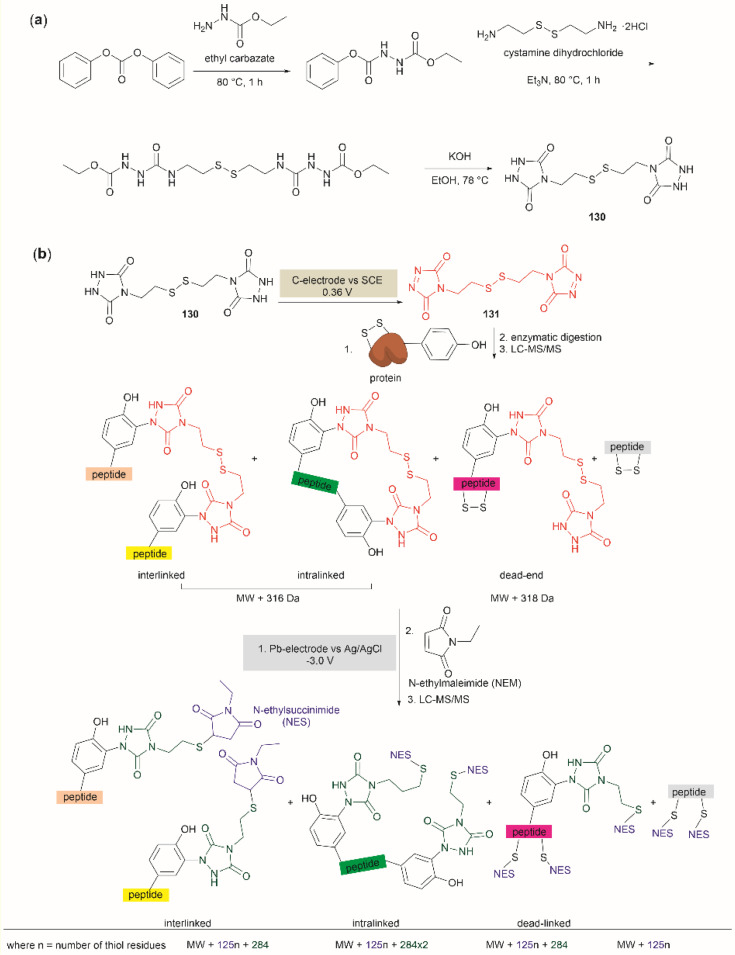
(a) Synthetic protocol for the preparation of DBB. (b) Process for structural analysis of proteins using the electrochemically activated DBB for Tyr-specific cross-linking reaction and subsequent disulfide bond cleavage.

Taking advantage of the selective labeling of Tyr *via* the electrochemical conversion of urazoles to 1,2,4-triazole-3,5(4*H*)-diones (TAD),^47^ together with the capability of electrochemical reduction of disulfide bonds, Cui *et al.*^136^ designed and synthesized [4,4′-(disulfanediyl bis(ethane-2,1-diyl))bis(1,2,4-triazolidine-3,5-dione)] (DBB, 130) as a Tyr selective protein cross-linker (Fig. 33a). The protocol for protein cross-linking with DBB involves the electrochemical oxidation of the N–N bond of the urazole 130 to a N=N bond in 131 under controlled potential (0.36 V), with subsequent Tyr-specific protein cross-linking. The protein is then subjected to enzymatic digestion and the digest is analyzed further by LC-MS/MS (Fig. 33b). At the end of this process, one can identify three types of cross-linked products including dead-end, interlinked and intralinked fragments, by analyzing mass differentials between the cross-linked processed proteins and the unmodified counterparts (Fig. 33b). However, one cannot discern between inter- and intralinked fragments. To achieve this differentiation, the cross-linked protein or peptide digested products are electrochemically reduced under an applied potential of −3 V, which breaks the disulfide bonds, including those natively present in the peptide fragments (or protein) and the ones in the linked DBB units. Subsequently, the mixture is treated with *N*-ethylmaleimide to cap the free thiol groups and the resultant products are analyzed further by LC-MS/MS. At this point one could determine which fragments were intralinked and which ones were interlinked.

This protocol was successfully applied for structural analysis of proteins, such as β-casein, r-hGH and BSA. Additional to sequence determination, with this method one can also infer some spatial information with regards to the inter- and intra- linked identified peptide fragments, as in principle the distance separating the linked Tyr residues is dictated by the length of the linker and driven by its conformational space. Thus, Cui *et al.* estimated that the distance separating inter- and intra- linked Tyr residues is constrained to ∼30 Å, which was calculated by adding the length of DBB (11.6 Å), the size of the Tyr side chain (4.6 Å), plus a variation attributed to protein flexibility. Another important finding was that DBB showed high selectivity for Tyr since no reactivity with Lys or Cys was detected.^136^

**Fig. 34:**
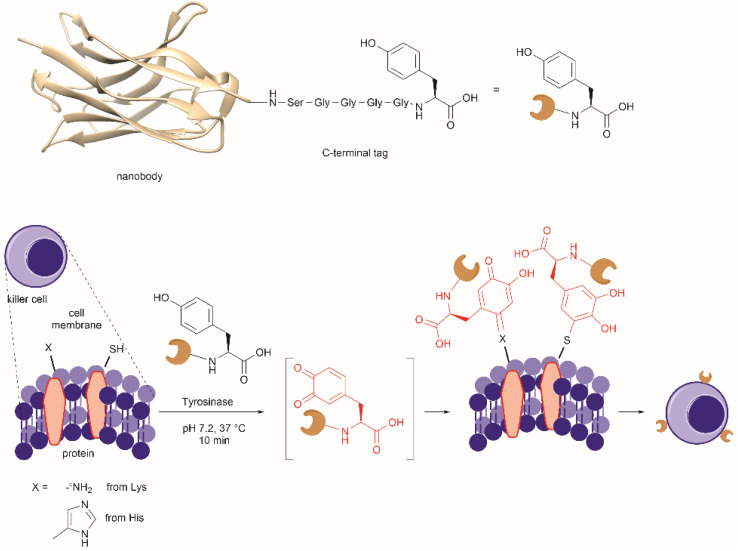
Protocol for Tyr-site-specific bioconjugation of nanobodies (PDB ID: 3K1K) to cell membranes.

A tyrosinase induced modification of cell surfaces was recently applied in the preparation of nanobody-cell conjugates.^137^ The methodology consisted of using abTYR, which has been shown to specifically oxidize Tyr located at the C-terminal end of proteins, thus promoting the conjugation between the resulting *ortho*-quinones and nucleophiles on the conjugation partner.^124^ Proteins lacking a C-terminal Tyr are genetically engineered to express a Tyr tag (*e.g.*, Ser–Gly_4_–Tyr). The model system of this application comprised: (1) Tyr C-terminal-tagged nanobodies, which are small antigen binders obtained from the variable regions of camelid immunoglobulins, and (2) natural killer (NK) cells, which are cells of the innate immune system with cytotoxic effector functions. The general idea was to label the membranes of NK with nanobodies to generate a targeted immunotherapeutic conjugated system (Fig. 34). Conjugation was first accomplished by exposing the NK-92MI cell line (∼1 × 10^6^ cells) to a 10 μM solution of a C-terminal tagged (Ser–Gly_4_–Tyr) nanobody known to recognize a green fluorescent protein in the presence of tyrosinase (400 nM) for 10 min at 37 °C. Under these conditions, it was determined that the modified NK cells remained viable and that the nanobodies retained their antigen recognition. Further, studies showed that the nanobodies remained bound to the cell surface with a half-life of ∼7.8 h, a value that is half of other comparable systems reported in the literature.^138^ It was also demonstrated that Lys, His and Cys residues present in the cell membranes were the nucleophilic centers for conjugation to the oxidized Tyr residues.

Additional experiments showed that under the above cited conditions ∼120 000 copies of nanobody were attached to the NK-92MI cell surface. Considering that nanobodies have a low molecular-weight of 10–15 kDa, one can depict a nanobody as an spherical particle of ∼3 nm diameter.^139^ Hence, one nanobody will have a circular area of ∼7.1 × 10^−6^ μm^2^, which will translate to ∼0.9 μm^2^ for 120 000 nanobodies. NK cells have a 6–7 μm diameter, which correspond to a ∼154 μm^2^ spherical surface area. With these numbers one can estimate that ∼0.6% of the surface of NK cells will be covered with nanobodies. This coverage value can be set as baseline associated with conjugation, cell viability and functionality as conjugated systems prepared with higher concentration of nanobodies (20 μM) were reported to lose activity. Data of comparable systems is scarce in the literature. For instance, Frank, *et al.* reported an antibody-cell conjugation method based on the modification of cell surfaces with a single-strand DNA functionalized with a succinimidyl ester; where the modified cells are further annealed with the complementary strand-modified antibody.^138^ The method was applied to the attachment of rituximab or daratumumab to cytokine-induced killer (CIK) cells, but antibody surface coverage was not determined.

NK-92MI cells were further conjugated to a nanobody that binds the human epidermal growth factor receptor 2 (HER2) tagged with the same C-terminal Ser–Gly_4_–Tyr fragment (nbHER2Tyr). It was shown that nbHER2Tyr-NK-92MI cells established specific contacts with HER2+ cells, and that only conjugated cells caused lysis of HER2+ SKBR-3 cells with no effect observed for HER2+ cells treated only with nbHER2Tyr or a mixture of nbHER2Tyr and the tyrosinase.

**Fig. 35:**
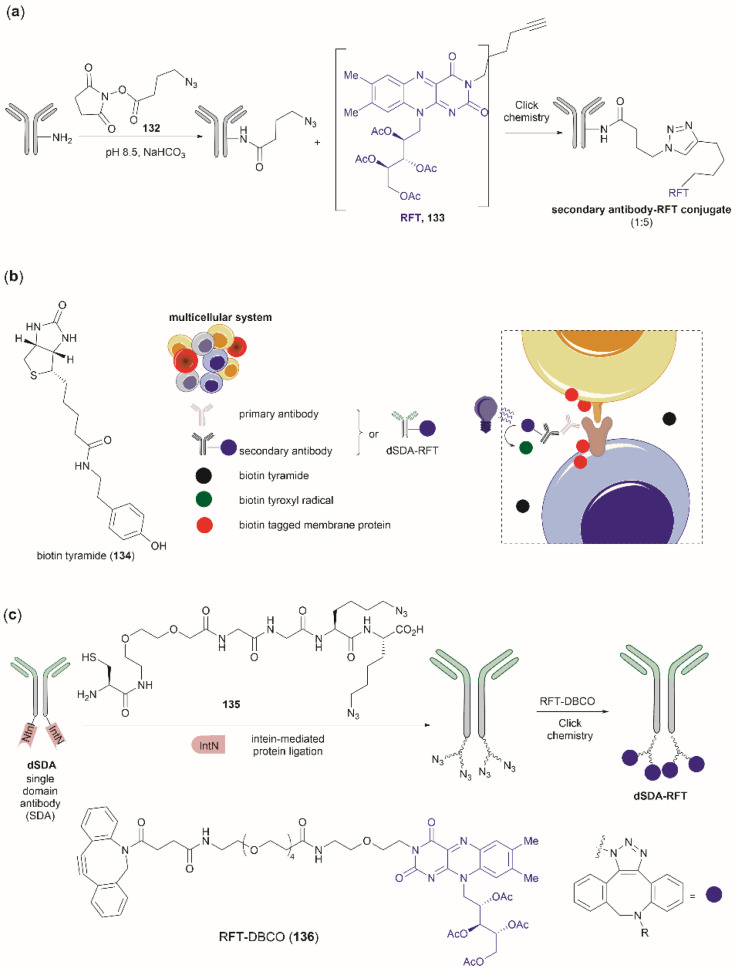
(a) The secondary antibody–flavin conjugate was prepared by non-selective labeling of lysine residues with azidobutyric acid NHS ester, followed by click reaction with an alkyne-RFT to yield an approximate 1 to 5 ratio of antibody to RFT. (b) Flavin-based photocatalytic Tyr site-selective tagging of proteins on cell surface. (c) SDA is expressed as an Fc–intein fusion for C-terminal attachment of the bis azide linker azido linker *via* intein-mediated ligation to form the dSDA homodimer. RFT–DBCO is covalently attached through a click reaction to the dSDA at the azido linkers to generate dSDA-RFT.

As mentioned above a flavin photocatalytic cell tagging method was developed to assess immune cell–cell interactions.^140^ The basic principle of this approach was to use the small molecule flavin cofactor to oxidize a cell-tagging phenol species, when it was near a cell–cell interface. The formed phenoxy radical then reacts with Tyr residues present in the proteomic synaptic cell–cell space, allowing to discriminate cell–cell interactions in a complex multicellular milieu. The initial implementation of the method comprised a primary/secondary antibody tagging system, where the primary antibody binds a specific cellular protein, and the secondary antibody recognizes the primary antibody. Importantly, the secondary antibody was derivatized with azidobutyric acid NHS ester 132 in a non-selective manner and then conjugated with riboflavin tetraacetate derivative 133*via* alkyne–azide click chemistry (Fig. 35a). Thus, the secondary antibody allowed the targeted delivery of the flavin unit. When cells were exposed to the antibodies in presence of biotin tyramide (134) and irradiated with blue light, tyramide was oxidized to the phenoxyl radical and reacted with tyrosyl radical of proteins near the tagging zone (Fig. 35b and 30). Important findings of this methodology were that: (1) while labeling was predominantly performed in Tyr residues, tagging of His and Trp also occurred, (2) the extent of protein biotinylation could be tuned by modifying the catalyst concentration, light duration and intensity, and (3) the extent of capture of trans-cellular interactions, when the ligand and its receptor were expressed in two different cells,^141^ was dependent on the macromolecular size of the tagging antibodies as points where protein–protein interactions occur present constrained intermembrane distances.

In a second approach the primary/secondary antibody tagging system was substituted with a single domain antibody (SDA), which recognizes the cell target protein without disrupting its interaction with its receptor. This SDA was expressed at the N-terminus of a human IgG crystallized fragment (Fc) to generate a dimeric form (dSDA) (Fig. 35c). Thus, the resulting fused single antibody system offered a size reduction of the tagging unit. Intein-mediated protein ligation was then used to site-specifically derivatize the fused system with a bis-azide linker 135,^142^ which was finally conjugated to a riboflavin derivative 136*via* dibenzylcyclooctyne (DBCO) copper free click chemistry. With the combination of the described Tyr biotinylation tagging technology and the use of oligonucleotide-barcoded streptavidin it was possible to capture uniquely interacting T cell populations from a mixed cell population of human peripheral blood mononuclear cells (PBMCs) and Raji cells.

**Fig. 36:**
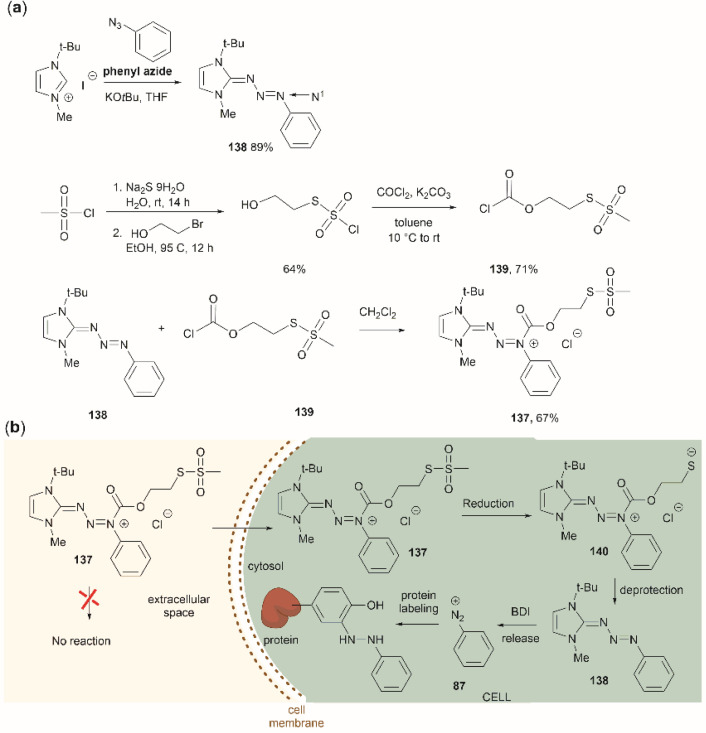
(a) Synthesis of triazabutadiene 137 with a protecting group cleavable under reducing conditions. (b) Proposed mechanism for the intracellular release of benzene diazonium ions (BDI) by 137.

Cornejo *et al.* designed and synthesized triazabutadiene compound 137 (Fig. 36a) that can release benzene diazonium ions (BDI) 87 intracellularly with spatial control upon exposure to the reducing intracellular environment (Fig. 36b).^143^ The bases for the structural design of compound 137 relied on the covalent protection of the N1 position of the triazabutadiene as a way to control the release of benzene diazonium ions. The authors of this work selected a sulfonyl thiolate as a protecting moiety responsive to intracellular reducing conditions in analogy to a disulfide group. The authors further posited that the positively charged small molecule would be able to cross the cellular membrane. Thus, the synthesis of 137 was readily accomplished in modest overall yields by reacting triazabutadiene 138 with chloroformate derivative 139 (Fig. 36a). A important feature of triazabutadiene 137 is its high stability in neutral aqueous media.^144^ An interesting result from this work is that the authors had hypothesized a decrease of protein phosphorylation in cells exposed to 137 and that this would be an indication of intracellular delivery, as it has been shown that derivatized Tyr residues are resistant to phosphorylation. Contrary to their hypothesis an increase of global tyrosine phosphorylation was observed for cells treated with 137, which was attributed to inhibition of tyrosine phosphatases. However, other potential adverse cellular effects provoked by 137 were not discounted (*e.g.*, DNA damage). Additionally, it was also shown that the reactivity of 137 is not specific to Tyr since azo derivatives with His were also detected. Despite the need to further probe the toxicity of 137 and similar derivatives, it is important to remark that this constitutes the first compound useful for intracellular delivery of benzene diazonium ions.

**Fig. 37:**
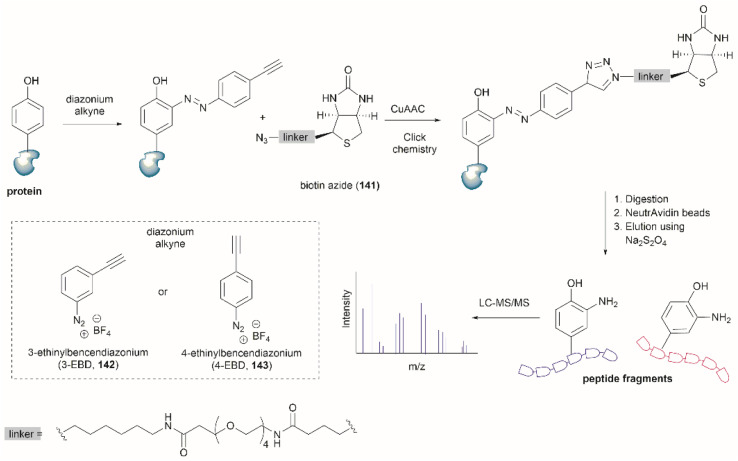
Protocol depiction for the comprehensive profiling of the tyrosine residues in the human proteome by integrating azo coupling and copper(i)-catalyzed azide alkyne cycloaddition (CuAAC).

Recently, a method to screen the reactivity of Tyr residues in human cell proteome (*e.g.*, cell lysates from MCF7) was developed.^145^ The method consisted of reacting proteins with an aryl diazonium-alkyne reagent, which results in 3-azo benzene derivatization of Tyr residues. Copper(i)-catalyzed azide alkyne cycloaddition was subsequently used to introduce a biotin tag 141, whose function was to capture Tyr labeled peptide fragments after protein digestion. Treatment of peptide enriched fractions with sodium dithionite generated the *ortho*-amino Tyr containing peptides, which are easily identified by MS (Fig. 37). Two bench-stable diazonium-alkyne compounds were studied in this work, 3-ethynylbenzendiazonium (142) and 4-ethynylbenzendiazonium (143), both as their corresponding tetrafluoroborate salts. These compounds presented different efficiency in labeling Tyr residues, where azo group formation was less efficient with 142, but both compounds were highly selective towards Tyr. Independent reports indicated that 142 predominantly reacted with Cys residues *via* a radical-based coupling mechanism,^146^ contradicting the findings of this work. Tyrosine quantification and protein functional correlations were accomplished using probe concentration-dependent experiments where two concentrations of 143, 500 μM for the high concentration (*H*) or 50 μM for the low concentration (*L*), were used in the labeling method. Over 5000 Tyr sites were identified with the reported method with 34% of the sites having *H*/*L* ratios of 10–20, 49% with *H*/*L* > 20, 17% had ratios of <10, among which only 9% had *H*/*L* ratios < 5. Proteins having relevant bioactivities (*e.g.*, catalytic or binding) were found in the group with *H*/*L* < 10. In particular, the quantified tyrosine residues with a *H*/*L* ratio < 5 (having higher Tyr reactivity) were found to be located in proteins presenting a wide variety of functions.

Interestingly, the Tyr quantification results of this study did not seem to agree with the ones performed previously where Tyr residues were first labeled *via* a sulfur–triazole reagent exchange method.^147^ This difference was attributed to different reaction mechanisms (electrophilic aromatic substitution for 142*versus* nucleophilic substitution for the sulfur–triazole compound) which might target different tyrosine residues in the proteome. However, it is important to highlight that comparison of both methodologies is further complicated by the fact that the sulfur–triazole study used HEK283T derived proteome and the high and low probe experimental concentrations (250 and 25 μM) were half of the concentration used in the aryl diazonium-alkyne tagging study. Overall, the applications discussed enable a better understanding of the functions of Tyr in relation to its chemical reactivity in proteins.

**Fig. 38:**
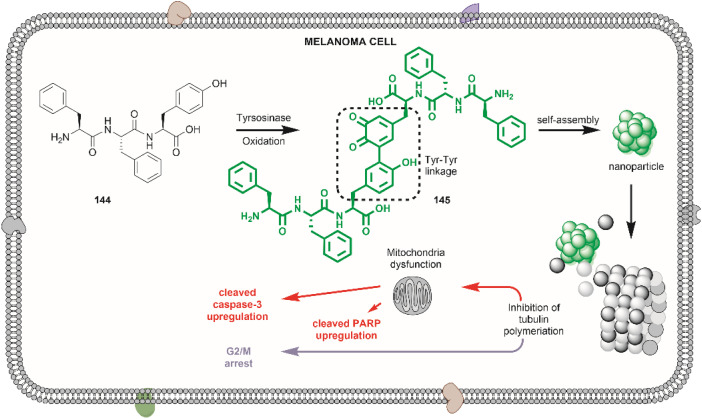
Phe–Phe–Tyr nanoparticle intracellular assembly upon oxidation with tyrosinase and the nanoparticle apoptosis effect against melanoma cells.

Malignant melanoma is characterized by an excess of melanin production by tyrosinase-catalyzed overoxidation. This particular trait of melanoma makes an attractive target for a targeted therapeutic interventions as conventional chemotherapeutic treatments (*e.g.*, cisplatin) have a high probability of developing resistance. Sun *et al.* reported tripeptide (Phe–Phe–Tyr, 144) which self-assembles into nanoparticles *via* formation of peptide Tyr-quinone dimer 145 after tyrosinase induced intracellular oxidation. The assembled structures presented intrinsic green fluorescence, a feature that allows determination of the integrity of the nanostructure intracellularly. Furthermore, the peptide nanoparticles were shown (1) to disrupt cytoskeleton formation by selectively interfering in tubulin self-polymerization, driving high levels of G2/M arrest, and (2) to obstruct mitochondrial function, inducing high levels of apoptosis factors (cleaved caspase 3 and cleaved poly ADP-ribose polymerase) (Fig. 38). These combined effects ultimately caused apoptosis against drug-resistant melanoma.^148^ The *in vivo* application of the peptide nanoparticle system was demonstrated by a twice peritumoral injection of the peptide (8.0 mg kg^−1^) in mice with a solid resistant melanoma tumor model with a volume of 150 mm^3^. Dynamic analysis of the tumor volume showed a reduction of 87.4% after treatment with the peptide, thus establishing a new path for biopharmaceutical targeting drug-resistant cancers. Moreover, an understanding of the assembly mechanism of the dimeric peptide and improvements in its solubility in water are aspects to consider for future applications of similar Tyr-specific dimerized peptide systems.

Sato *et al.* reported the fast preparation of antigen responsive-fluorogenic turn-ON immunosensors following a two-stage process. The first step involves the selective modification of Tyr residues located in the antibody complimentary determining region (CDR) with an azide containing compound and the second step included the introduction of a fluorescent dye using Cu-free click chemistry reaction.^149^ Important requirements for the immunosensor application are that the chemical modifications do not alter the antigen-binding site, that the fluorescent molecule is located near the antigen-binding site and the fluorescence is quenched due to photoinduced electron transfer of near aromatic residues (*e.g.*, Trp) in the antigen-free state.

**Fig. 39:**
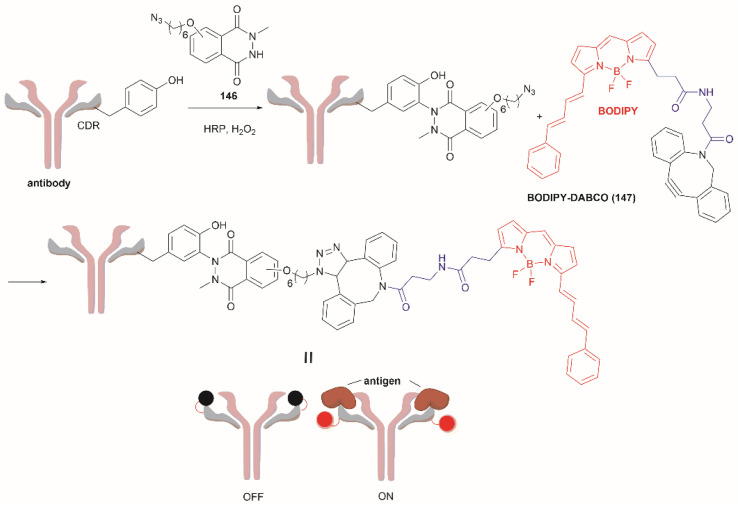
Methodology for the preparation of antigen responsive-fluorogenic turn-ON immunosensors.

Sato *et al.* demonstrated the applicability of their method with the full-length pharmaceutical antibodies, trastuzumab and rituximab. The authors used an azide-conjugated *N*-methylated luminol derivative 146 previously reported by their group^150^ for Tyr-selective antibody modification, and then screened a series of DABCO functionalized fluorescent molecules by introducing them into the azide-modified antibody *via* a Cu-free click reaction (Fig. 39). BODIPY dyes (*e.g.*, 147) showed the best fluorescence response during antibody denaturation and antigen addition tests (turn-ON conditions) (Fig. 39). This was attributed to the hydrophobic characteristics of the dyes, which presumably favors the dyes binding to the internal structure in CDR, resulting in efficient PET quenching by Trp residues.

A word of warning from Sato and collaborators is in relation to the generality of their method where the Tyr residue in the CDR should not be essential in antigen recognition as its modification might lead to an inactive antibody. Importantly, the method reported by Sato *et al.* enables the fast screening of fluorescent dyes and provides an important alternative to genetic engineering methodologies for the rapid preparation and evaluation of biosensors.

There are also recent examples on the applications of genetically encoded Tyr-functionalized residues in proteins. An oxygen tolerant alcohol dehydrogenase II was engineered by the site-specific introduction of l-DOPA into the wild-type enzyme. The mutated enzyme presented a strong binding affinity for Zn^2+^ and was shown to be a potential candidate system for biofuel production in photosynthetic organisms in presence of oxygen.^151^ Furthermore, the utilization of genetically encoded l-DOPA for protein labeling *via* strain-promoted labeling induced by oxidation with tyrosinase has been reported.^152^

## 5 Conclusions

Given the biological significance of Tyr and its post-translationally modified analogues, demands for tools that allow selective modification of Tyr within peptides/proteins are surging in recent years. This pursuit has been further driven by the unique chemical, electrochemical and photochemical properties of Tyr, which have led to the development of a wide array of Tyr-selective modification techniques. While some methods (such as C–H activation of Tyr) have exhibited low residue selectivity and harsh conditions, these modifications of Tyr are still of key interest for medicinal chemists to explore structural diversification of Tyr during peptide drug optimization. More importantly, Tyr chemistry exhibiting high selectivity and compatibility with protein substrates has garnered increasing interest due to its immense potential in applications such as protein labeling, targeted drug delivery, and biomaterial development. Compared to other proteinogenous amino acids (*e.g.*, Cys and Lys), Tyr exhibits both relatively low abundance on protein surfaces and is uncharged at physiological pH. These factors are important to minimize disruption of protein/peptide activity after protein modification. Moreover, highly homogenous modification/bioconjugation at Tyr sites could be realized as the majority of Tyr residues are inaccessible to exogenous reagents and a single solvent-exposed Tyr tag can be usually installed on the protein termini *via* protein engineering (such as tyrosinase-mediated oxidative coupling). These favorable properties have rendered Tyr a desired target for protein conjugation and a viable alternative to Cys and Lys. Furthermore, Tyr-selective modification of proteins can also be performed using genetic code engineering technologies, albeit issues like deficient uptake of the non-canonical amino acid and/or poor protein incorporation still need to be addressed in the future.

One overarching goal of Tyr-selective modification namely peptide/protein cleavage at Tyr sites, has long been an underestimated transformation which also finds an array of promising applications (*e.g.*, proteomics and site-specific functionalization). A variety of chemical, enzymatic and electrochemical means have demonstrated the ability to cleave peptides selectively at Tyr sites, thus enabling facile determination of the primary structure of unidentified protein/peptides and even complex cyclic peptides. It is interesting to note that peptide fragments bearing selectively modified C- or N- moieties derived from Tyr cleavage can also be further functionalized thus providing a latent conjugation/modification site at peptide termini. Taking into account the recent advancements in the field of Tyr-selective chemistry, we anticipate a rapid rise in the development of more robust Tyr-targeting modification techniques, as well as the widespread application of these technologies in practical settings, such as the development of antibody-drug conjugates.

## Data availability

This review article does not include any experimental or computational data.

## Author contributions

S. Z. and L. M. D. L. R. conceived the idea. S. Z., L. M. D. L. R., F. F. L. and M. A. B. wrote the manuscript with input from all authors. All authors have given approval to the final version of the manuscript.

## Conflicts of interest

There are no conflicts to declare.

## Supplementary Material
